# Accelerated Intermittent Theta-Burst Stimulation for Treatment-Resistant Bipolar Depression: A Randomized Clinical Trial

**DOI:** 10.1001/jamanetworkopen.2024.59361

**Published:** 2025-02-11

**Authors:** Lawrence G. Appelbaum, Hadley Daniels, Liam Lochhead, Bianca Bacio, Robin Cash, Cory R. Weissman, Jordan N. Kohn, Itay Hadas, Zafiris J. Daskalakis

**Affiliations:** 1 Department of Psychiatry, Interventional Psychiatry Program, University of California San Diego, La Jolla; 2 Department of Biomedical Engineering and Department of Psychiatry, University of Melbourne, Melbourne, Victoria, Australia

## Abstract

This randomized clinical trial evaluates the effect of accelerated intermittent theta-burst stimulation, compared with sham stimulation, on depression severity in individuals with treatment-resistant bipolar depression.

## Introduction

Bipolar depression (BD) is a serious mental illness with few effective therapies. Sheline and colleagues^1^ recently reported findings from a randomized, sham-controlled trial testing the efficacy of an accelerated 5-day schedule of intermittent theta-burst stimulation (aiTBS) in 24 patients with treatment-resistant BD. They found that active aiTBS was significantly more effective than sham stimulation for depressive symptoms, supporting the clinical efficacy of accelerated transcranial magnetic stimulation in individuals with BD. We present evidence from a randomized double-blind sham-controlled clinical trial conducted in 13 patients with BD.

## Methods

This preregistered (NCT05393648) clinical trial, conducted between July 21, 2022, and March 8, 2024, followed the CONSORT guideline and was approved by the University of California, San Diego institutional review board. The trial protocol is available in Supplement 1; detailed methods are provided in the eMethods in Supplement 2. Patients aged 18 to 70 years who provided written consent, were diagnosed with bipolar I or II, and were experiencing a current depressive episode were eligible (eTable 1 and eFigure in Supplement 2). Participants were required to have treatment-resistant BD and Montgomery-Åsberg Depression Rating Scale (MADRS) scores above 19 at screening. Participants underwent anatomical and resting-state functional connectivity magnetic resonance imaging scans to compute personalized left dorsolateral prefrontal cortex targets (eTable 2 in Supplement 2) by connectivity to subgenual anterior cingulate cortex using seedmap and clustering methodology.^2^ Stimulation targeting was optimized with electric-field modeling.^3,4^ Patients received 10 hourly, 9-minute sessions of imaging-guided aiTBS for 5 days at 90% resting motor threshold. Coil position was maintained using neuronavigation, and placebo was delivered using somatosensory-matched sham stimulation. The primary outcome was change in MADRS scores from pretreatment to 1-week follow-up. Response was defined as at least a 50% reduction in scores. Robust linear mixed effects modeling was used to evaluate treatment effects between and within groups, and questionnaires were used to assess masking quality (eTable 3 in Supplement 2). R studio, version 4.3.1 (R Foundation), was used for analysis, and a 2-sided *P* < .05 was considered significant.

## Results

Eighteen participants were recruited and 13 were randomized (5 active, 8 sham), of whom all completed the full treatment course (Table). Mean (SD) MADRS scores in the active group were 30.2 (9.5) at baseline, 14.0 (11.4) at 1 week, and 14.0 (12.8) at 4 weeks, with 60% (3 of 5) achieving treatment response (Figure, A). Mean (SD) MADRS scores were 29.0 (4.8), 24.6 (9.9), and 22.4 (12.1) at these time points for sham participants, with 12.5% (1 of 8) responding. There were no significant between-group differences in MADRS scores at 1 week (difference, 10.52; *d* = 1.24 [95% CI, −0.23 to 2.71]; *P* = .10) or 4 weeks (difference, 9.58; *d* = 1.13 [95% CI, −0.37 to 2.64]; *P* = .14). However, the active group MADRS scores were significantly lower at 1 week (difference, −16.14; 56.8% improvement; *d* = 1.91 [95% CI, 0.64-3.18]; *P* = .006) and 4 weeks (difference, −16.57; 55.2% improvement; *d* = 1.96 [95% CI, 0.69-3.23]; *P* = .005) than at baseline (Figure, B), whereas sham group scores did not differ (1-week difference, −4.22; 12.2% improvement; *d* = 0.50 [95% CI, −0.51-1.50]; *P* = .52; 4-week difference, −5.59; 20.0% improvement; *d* = 0.66 [95% CI, −0.39-1.71]; *P* = .37). No participants reported hypomania or mania throughout the study as measured by the Young Mania Rating Scale.

**Table.** Baseline Patient Characteristics for Each Treatment Group

Baseline characteristic^a^	Participants, No. (%)	
	Sham (n = 8)	Active (n = 5)
Age, mean (SD), y	41.75 (19.3)	49.8 (10.1)
Sex		
Female	4 (50)	4 (80)
Male	4 (50)	1 (20)
Race and ethnicity		
African American or Black	1 (12)	0
Hispanic or Latino	3 (38)	2 (40)
White	4 (50)	3 (60)
Educational attainment, mean (SD), y	15.7 (1.8)	15.8 (2.3)
Diagnosis		
Bipolar II	3 (38)	2 (40)
Bipolar I	4 (50)	0
Data not available	1 (12)	3 (60)
Trials (mean SD), No.		
Antidepressant	5.0 (1.8)	5.6 (1.5)
Augmentation	0.6 (0.8)	1.0 (0.7)
Previous pharmacotherapy trials		
Lithium	3 (38)	3 (60)
Anticonvulsants	7 (88)	3 (60)
SSRIs	4 (50)	4 (80)
SNRIs	3 (38)	3 (60)
Other antidepressants	4 (50)	3 (60)
Atypical antipsychotics	4 (50)	3 (60)
Concomitant pharmacotherapy		
Lithium	1 (12)	2 (40)
Anticonvulsants	5 (63)	4 (80)
SSRIs	3 (38)	1 (20)
SNRIs	1 (12)	1 (20)
Other antidepressants	3 (38)	2 (40)
Atypical antipsychotics	3 (38)	4 (80)
Psychiatric comorbidities		
Anxiety	6 (75)	1 (20)
Eating disorder	1 (13)	1 (20)
ADHD	2 (25)	0
Medical comorbidities		
Hypothyroidism	2 (25)	1 (20)
Migraine	3 (38)	1 (20)
Hypertension	1 (13)	1 (20)
Multiple Sclerosis	1 (13)	0
GERD	2 (25)	0

Abbreviations: ADHD, attention-deficit/hyperactivity disorder; GERD, gastroesophageal reflux disease; SNRI, serotonin-norepinephrine reuptake inhibitor; SSRI, selective serotonin reuptake inhibitor.

^a^ Age, sex, race and ethnicity, and educational attainment were self-reported by the patients. Race and ethnicity are reported because they may be associated with bipolar depression severity and treatment response.

**Figure.**
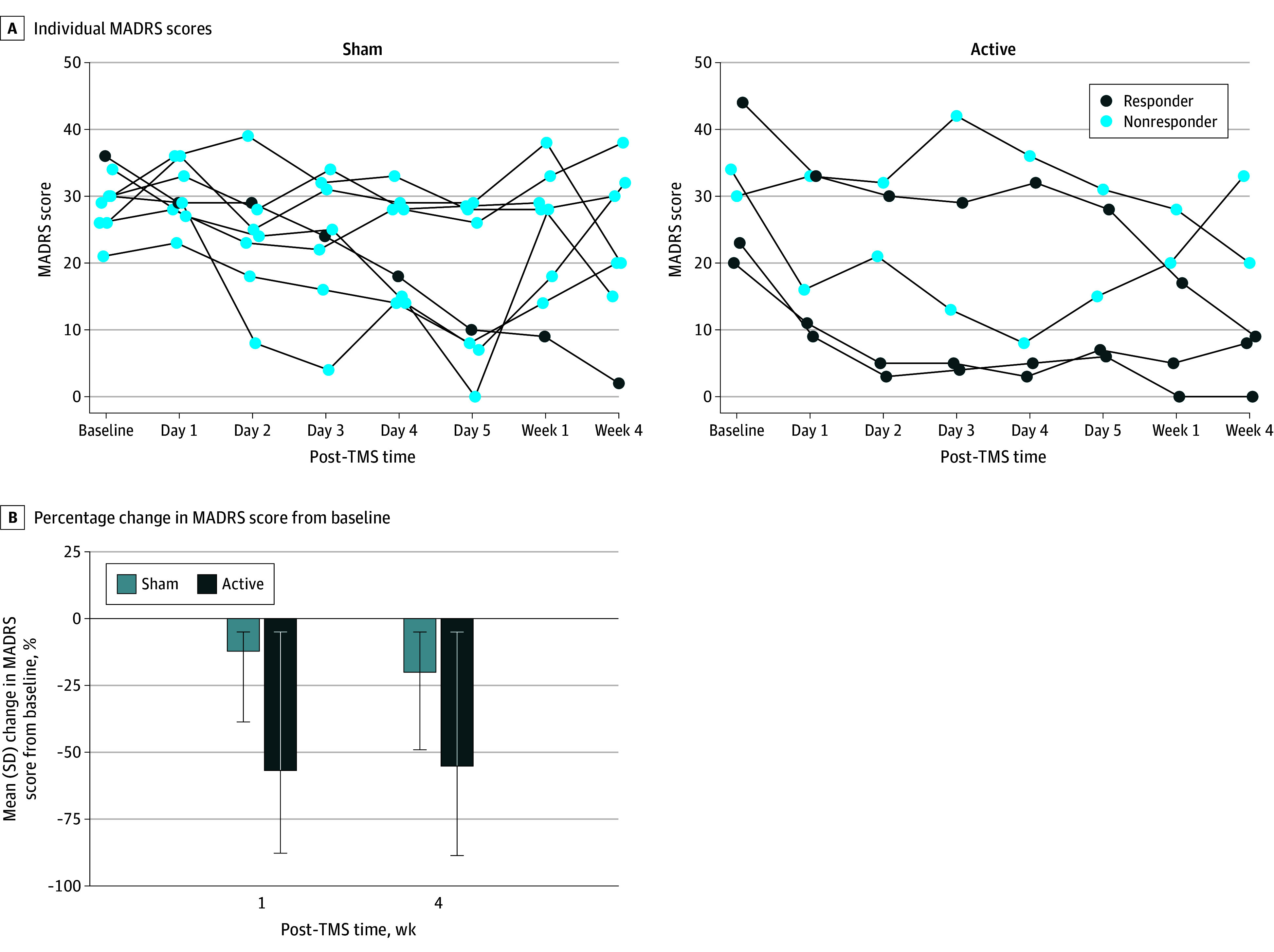
Raw Montgomery-Åsberg Depression Rating Scale (MADRS) Scores and Change in Scores From Baseline TMS indicates transcranial magnetic stimulation.

## Discussion

This study found similar but smaller positive effects as reported in Sheline et al,^1^ providing corroborating evidence to support the rapid clinical benefits of connectivity and e-field optimized aiTBS for treatment-resistant BD. Within-group improvements for active aiTBS indicated a significant and sustained reduction in depression severity at 1 and 4 weeks. Several innovations may contribute to these effects, including the accelerated schedule, personalized targeting,^5^ and e-field dose optimization.^4^ Limitations included the small sample size and unbalanced group demographics. Future studies should aim to expand on these promising results.

## Notes

Supplement 1.
Trial Protocol

Supplement 2.
eMethods.
**eFigure.** CONSORT Flow Chart **eTable 1. **Inclusion/Exclusion Criteria **eTable 2.** Target x, y, and z Coordinates and Coil Orientation Used for Treatment **eTable 3.** Guesses, Confidence, and Actual Treatment Assignments
eReferences.

Supplement 3.
Data Sharing Statement
