# Supplementary material for: Accelerated Intermittent Theta-Burst Stimulation for Treatment-Resistant Bipolar Depression: A Randomized Clinical Trial
Source: JAMA Netw Open. 2025 Feb 11;8(2):e2459361. doi: 10.1001/jamanetworkopen.2024.59361 (PMC11815521; doi:10.1001/jamanetworkopen.2024.59361)
Supplement: Supplement 1. — Trial Protocol [file jamanetwopen-e2459361-s001.pdf]

UCSD Human Research Protections Program
New Biomedical Application
RESEARCH PLAN

Instructions for completing the Research Plan are available on the HRPP website.
The headings on this set of instructions correspond to the headings of the Research Plan.
General Instructions: Enter a response for all topic headings.
Enter “Not Applicable” rather than leaving an item blank if the item does not apply to this project.

Version date: 9/30/2013

1. PROJECT TITLE

Bipolar efficacy biomarkers for accelerated intermittent theta burst rTMS trial

2. PRINCIPAL INVESTIGATOR

PI: Itay Hadas, PhD

Collaborators:
Zafiris Jeff Daskalakis, MD, PhD
Lawrence Gregory Appelbaum, PhD
Weissman Cory, MD
Yvette Sheline, MD (UPenn, CNDS)
Claudia Baldassano, MD (UPenn, CNDS)
Michael Thase, MD (UPenn, CNDS)

3. FACILITIES

UCSD Interventional Psychiatry Clinic (IPC) site:
4S Health Center
16918 Dove Canyon Road, Suite 100
San Diego, CA, US, 92127
MC 8322

UC San Diego Center for Functional MRI:
9500 Gilman Drive
La Jolla, CA 92093
MC 0677

Center of Neuromodulation in Depression & Stress (CNDS), University of Pennsylvania (UPenn)
3700 Hamilton Walk, 3rd Floor
Philadelphia, PA 19104-4283

4. ESTIMATED DURATION OF THE STUDY

1 year after IRB approval.

5. LAY LANGUAGE SUMMARY OR SYNOPSIS (no more than one paragraph)

The research study is being conducted to test whether using high dose spaced theta-burst rTMS (a form of repetitive transcranial magnetic stimulation) produces a significant reduction in depressive symptoms compared with sham. This project will recruit patients aged 18-70 with symptoms of bipolar depression (BPD) who have failed (or not shown signs of improvement) after at least two prior treatments. The null hypothesis is that there will be no difference in reductions in depressive symptoms by the end of a five-day treatment period. The alternative hypothesis is that, compared with sham, active TMS will result in a greater reduction in depressive symptoms by the end of the treatment period.

To facilitate the development of rTMS protocols there is a need for biomarkers that are sensitive to BPD symptom severity and clinical improvement. Previously in our lab, we developed biomarkers suitable for depression trials, and these biomarkers are very likely to show sensitivity to BPD, since they are associated with brain regions and functions associated with BPD. As a secondary aim, we will try to identify biomarkers in cortical region associated with BPD, and formulate a statistical model that may be able to predict BPD remission after the treatment. this study will lead to development of new brain stimulation treatment protocols and biomarkers, will aid in treatment selection, and eventually lead to better clinical outcome for patients suffering from BPD.

6. SPECIFIC AIMS

Aim 1: To assess the clinical efficacy, safety, and tolerability of compared to sham stimulation (i.e., placebo) for BPD at two clinical sites (UCSD-IPC and UPenn-CNDS).

Aim 2: To assess the engagement of transcranial magnetic stimulation concurrent with encephalogram (TMS-EEG) (at UCSD-IPC) and resting state functional magnetic resonance imagining (rs-fMRI) (at UCSD-IPC and UPenn-CNDS) BPD biomarkers with compared to sham treatment.

7. BACKGROUND AND SIGNIFICANCE

The primary aim of this proposal is to assess the therapeutic efficacy of , a novel treatment protocol for bipolar disorder. The secondary goal of this proposal is to use transcranial magnetic stimulation (TMS) combined with electroencephalography (EEG) to evaluate subgenual cingulate cortex (SGC) – dorsolateral prefrontal cortex (DLPFC) connectivity. This brain circuitry metric is associated with BPD pathophysiology and will be used to assess BPD severity and predict treatment efficacy.

BPD is a leading cause of disability worldwide, it is characterized by recurring episodes of mania and depression (Carvalho et al., 2020). Psychotherapy and pharmacotherapy are effective, but BPD is remitting and relapsing chronically, and depression episodes are more prevalent and debilitating than mania occurrences throughout the life span of BPD patient (Konstantinou et al., 2021). Recent developments in brain stimulation and imaging technologies allow a more specific modulation of brain circuits that are implicated in psychiatric conditions. Devices such as rTMS can focally stimulate pathophysiological associated brain circuits and modulate their activity (Fox et al., 2012; Hadas et al., 2019). Apart from its anatomical focality, rTMS can produce temporally precise stimulation patterning that corresponds with brain physiological signal. In this manner, it is possible to alter a specific brain pathological circuit, and produce therapeutic effects even more effectively (Blumberger et al., 2018; Cole et al., 2020). Indeed, novel protocols that target pathophysiologically specific brain circuits were found to be efficacious in obsessive compulsive disorder (Carmi et al., 2018), posttraumatic stress disorder (Kan et al., 2020), ADHD (Alyagon et al., 2020; Hadas et al., 2021), addictions (Addolorato et al., 2017; Dinur-Klein et al., 2014), and most prominently, treatment resistant depression (Fitzgerald et al., 2006; Blumberger et al., 2018; Cole et al., 2020). Early evidence suggests that HDS-TBS may be the most effective rTMS protocol for treating depressive symptoms. Currently, several rTMS trials efficaciously treated BPD during depressive episodes (McGirr et al., 2016; Konstantinou et al., 2021). However, none of the rTMS-BPD trials utilized the novel HDS-TBS protocol (apart from Tischler et al., 2019, pilot trial with n=4).

SGC activity and connectivity is widely associated with emotional dysregulation (Drevets et al., 2008; Harrison et al., 2009), depression (Drevets et al., 1997; Fox et al., 2012; Hadas et al., 2019) and BPD (Hajek et al., 2008; Houenou et al., 2007). TMS-EEG is a reliable tool for assessing brain circuitry, it allows to experimentally control the stimulation timing and anatomical targeting, and can produce a highly reliable, localized activity and connectivity metrics. Previously developed SGC connectivity biomarker at our lab demonstrated sensitivity for depressive symptoms severity. This biomarker also adhered to symptom improvement after rTMS treatment, but

not after sham stimulation (Hadas et al., 2019). The overall aim of this study is to compare the clinical and physiological effects of HDS-TBS to sham HDS-TBS, and to develop TMS-EEG metrics for SGC connectivity as biomarkers for BPD severity and as predictors for clinical response in BPD. Our hypothesis is that HDS-TBS will produce significant clinical improvement in BPD compared to sham HDS-TBS. Additionally, we hypothesize that SGC connectivity biomarkers will correlate with BPD symptom severity, and will be able to predict BPD remission.

8. PROGRESS REPORT

This is a new study.

9. RESEARCH DESIGN AND METHODS

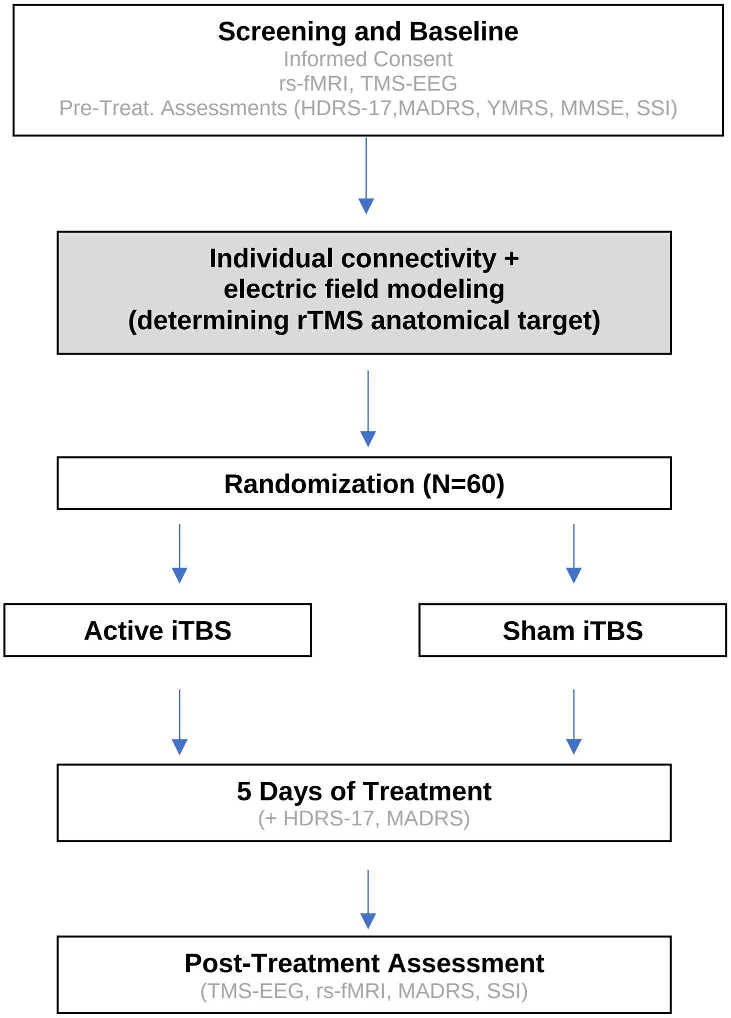

* TMS-EEG at baseline and post-treatment will be conducted at UCSD-IPC only. All other procedures stated in this protocol are identical at UCSD-IPC and UPenn-CNDS.

This study will be randomized, double blind and sham-controlled with HDS-TBS or sham HDS-TBS directed at the left DLPFC of BPD patients. Participants will be recruited and will receive the Structured interview for Diagnostic and Statistical Manual of Mental Disorders, 5th Edition (DSM-V), the Montgomery-Asberg Depression Rating Scale (MADRS), 17-item Hamilton Rating Scale for Depression (HRSD-17), The Beck Scale

for Suicide Ideation (SSI), Mini-International Neuropsychiatric Interview (M.I.N.I.), Antidepressant Treatment History Form (ATHF), Patient Health Questionnaire (PHQ-9), Generalized Anxiety Disorder Assessment (GAD-7), the Young Mania Rating Scale (YMRS), the Oldfield Assessment for Handedness (handedness will be included in data analysis but is not an exclusionary criterion), Mini-Mental State Examination (MMSE >24) and Demographic and MRI screening questionnaires to determine eligibility. Following informed consent, participants will have a structural MRI, and functional connectivity rs-fMRI scan. Additionally, participants will go through a TMS-EEG neurophysiological assessments. We will determine the TMS-EEG and rTMS coil positioning per participant, based on rs-fMRI co-activity anticorrelations between the SGC seed and left-DLPFC, in accordance with positioning algorithms described in the most recent literature (Balderston et al., 2022; Cash et al., 2021). After the baseline assessments, participants will receive double blind randomization to active treatment with HDS-TBS rTMS or sham treatment. Both active and sham treatment will consist of 5 days of HDS-TBS regimen (active and sham stimulation will be operationalized by the side of the coil directed at the participant head). During treatment course participants will be evaluated daily for MADRS and HRSD-17. At the end of each 5-day treatment patients will again receive an rs-fMRI scan, TMS-EEG measurement (only at UCSD-IPC site), MADRS, HRSD-17 and SSI clinical assessments.

Study participants will complete the following study procedures (TMS-EEG at baseline and post-treatment will be conducted at UCSD-IPC only. All other procedures will be conducted at UCSD-IPC and UPenn-CNDS).

Visit 1: Screening Visit
Informed consent will be obtained at the beginning of Visit 1 prior to any study-related procedures. Any questions or concerns will be addressed by study personnel prior to obtaining consent. A copy of the signed consent will be given to the participant; the investigators will retain the original consent. Screening assessments will include the M.I.N.I., ATHF, PHQ-9, GAD-7, HRSD-17, MADRS, SSI, YMRS, Oldfield Assessment for Handedness , MMSE, and Demographic and MRI screening questionnaires to determine eligibility.

Visit 2: Pre-treatment MRI Scan
Participants will undergo a 1 hour resting state fMRI scan session, including a T1 weighted anatomical MRI, a T2 weighted MRI optimized for hippocampus, a diffusion weighted MRI, and SGC seed based rs-fMRI (for TMS coil position optimization). All the MRI scans will take place at the UC San Diego Center for Functional MRI (9500 Gilman Drive, La Jolla, CA 92093, MC 0677) or UPenn-CNDS. Some of the clinical assessments might be conducted during this visit.

Visit 3: Pre-treatment TMS–EEG (neurophysiological assessment) (UCSD-IPC only)
will take place at the UC San Diego Health Interventional Psychiatry clinic (IPC). During Visit 3, TMS-EEG will be administered. It will consist of motor evoked potentials via electromyography (EMG), heart rate monitoring via electrocardiogram (ECG), and three TMS-EEG measurements (TEP, LICI and Sham). For each measurement, 100 TMS pulses (200 for LICI – a paired-pulse protocol) will be delivered. The coil will be directed at the optimal M1-ABP activation hot spot for MEPs, and to the left DLPFC (using rs-fMRI informed stimulation site) for TMS-EEG readings. The coil will be flipped above the DLPFC-determined hot-spot for sham (coil magnetic field is directed away from the participant's head). Visit 3 will last approximately 3 hours. Additionally, visit 3 will also include the following clinical assessments: MADRS, HRSD-17, PHQ-9, GAD-7, and SSI questionnaires. UPenn-CNDS will conduct these clinical assessments at visit 2.

Visits 4-8 (Treatment course): the will take place at the UC San Diego Health Interventional Psychiatry clinic (IPC) or UPenn-CNDS. During Visits 4-8, the participant will sit in a comfortable chair, A double sided

active/placebo TMS coil will be used. The coil's treatment side will be assigned ensuring blinding of both patient and technician. The coil will be positioned above individually targeted left-DLPFC hot-spot. The HDS-TBS treatment protocol involves delivery of 9 minutes and 40 seconds session, with 10 sessions daily, separated by 50 min break (10 hours/day), for 5 days, with rTMS pattern of 50 Hz pulse triplet bursts, delivered at a 5-Hz theta rhythm. The stimulation intensity will be 90% of RMT
The rTMS device for administering the

* UPenn-CNDS site will use FDA approved, class II, MagPro X100 (Magventure)

Visit 9: Post-treatment TMS–EEG (neurophysiological assessment) (UCSD-IPC only) and clinical assessments
This visit will be similar to visit 3 and will occur 1 week after the treatment end point. The visit will include motor evoked potentials via electromyography (EMG), heart rate monitoring via electrocardiogram (ECG), 3 TMS-EEG measurements (TEP, LICI and sham), and clinical assessments: MADRS, HRSD-17, and SSI questionnaires.

Visit 10: Post-treatment fMRI Scan.
This visit will be similar to visit 2 and will occur 1 week after the treatment end point.

Schedule of study visits

Procedures	Screening Visit 1	Baseline		Acute Phase (HDS-TBS /sham) Visits 4-8	Post 1-HDS-TBS /sham Visit 9	Post 2-HDS-TBS /sham Visit 10
		Visit 2	Visit 3			
Informed Consent	x					
Demographics	x					
Medical History (ATHF)	x					
SCID	x					
MADRS	x		x	x	x	
SSI	x		x		x	
YMRS	x					
Oldfield Assessment for Handedness	x					
MMSE	x					
HRSD-17	x		x	x	x	
Concomitant Meds	x	x	x	x	x	

AE/SAE Recording	x	x	x	x	x	
TASS	x					
Randomization		x				
MRI		x				x
RMT			x	x		
TMS-EEG (occurring at UCSD-IPC only)			x		x	

Magnetic Resonance Imaging (pre- and post-treatment). Participants will undergo a 1-hour MRI scan session, including a T1 weighted anatomical MRI, a T2 weighted MRI optimized for hippocampus, a diffusion weighted MRI, and SGC seed based rs-fMRI . The MRIs need to be 3D with sagittal orientation and 0mm gap between slices and 1mm thickness of slice. During the scanning, ears and nose should be included.

EMG (as part of pre- and post-treatment neurophysiological TMS-EEG visits). A surface EMG will record motor evoked potentials (MEP) induced by the TMS targeting the motor cortex. The EMG Ag-AgCl electrodes will positioned over the belly of the abductor pollicis brevis (APB) muscle. The EMG signal will be monitored on a computer screen. The signal will be amplified (Intronix Technologies Corporation Model 2024F, Bolton, Ontario, Canada) filtered (band pass 2Hz - 5kHz), digitized at 5 kHz (Micro 1401, Cambridge Electronics Design, Cambridge, UK) and stored in a laboratory computer for off-line analysis. The subjects will be instructed to relax throughout the study. Trials contaminated with voluntary muscle activity will be rejected.

ECG (as part of pre- and post-treatment neurophysiological TMS-EEG visits). A surface ECG will record heart rate. The ECG Ag-AgCl electrodes will positioned on each arm and the left ankle. The ECG signal will be monitored on a computer screen. The signal will be amplified via the Synamps 2 EEG system (Neuroscan, Compumedics, USA) and stored in a laboratory computer for off-line analysis.

TMS-EEG (neurophysiological visit – UCSD-IPC site only). TEPs (TMS evoked potential) will be recorded through a 64-channel Synamps II EEG system (Neuroscan, Compumedics, USA). 64 channel EEG cap will be fitted on the participant's head. Earbuds will be placed in the participants ears playing white noise (adjusted to conceal TMS clicking noise), ear-muffs will be placed on top of the ear buds. TMS will be delivered by using two Magstim-200 stimulators (Magstim Company Ltd., UK) and MEP data for the resting motor threshold (RMT) procedure will be collected using commercially available software, Signal (Cambridge Electronics, UK). The coil will be directed at the left DLPFC (using rs-fMRI informed stimulation site) utilizing the dedicated neuronavigation system. The TMS paradigms to be used include single pulse, long interval cortical inhibition (LICI) – paired pulse stimulation (Farzan et al., 2016). 100 pulses of sham stimulation protocol will be also administered, where the coil is facing away from the participant's head. The EEG signal will be processed using Matlab (The Mathworks Inc., USA) customized scripts for analysis of TEP, spectral perturbations, source localization and brain connectivity (Sun et al., 2016; Hadas et al., 2019; Hill et al., 2021). Analyses will proceed according to methods developed or established in our laboratory.

Localization of M1 (as part of pre-treatment neurophysiological TMS-EEG visits). The hot spot for MT determining (cortical representation of APB in M1) will be found by moving the coil around the scalp and using TMS intensities that can evoke MEPs of around 1mV over APB. When the largest MEPs with the corresponding APB movement are found at a specific coil position on top of the scalp, the coil orientation is optimized so it evokes the largest response at the given spot. Then the coil positioning and orientation is saved

in the neuronavigation system so it can be replicated for the MT calculation. If the neuronavigation system is not used the location is marked on the scalp with a removable mark (i.e., Sharpie).

Resting Motor Threshold (RMT, as part of pre-treatment neurophysiological TMS-EEG visits). The RMT is determined by placing the coil on the head targeting the cortical representation (hot spot) of a hand muscle (i.e., APB) and then evoking contralateral motor evoked potentials (MEP). The RMT is the minimum stimulus intensity that elicits an MEP of >50 mV in at least 5 out of 10 trials in the voluntarily relaxed right APB (Rossini et al., 2015).

Neuronavigation (as part of pre-treatment neurophysiological TMS-EEG visits; part of the first treatment visit at UPenn-CNDS). The system allow to localize the TMS coil in reference to the participants head, online – during the neurophysiological and treatment sessions. An MRI-guided neuronavigation system will be used on the first neurophysiological visit (first treatment visit at UPenn-CNDS) to determine the precise stimulation location, and coil orientation. The neuronavigation system is an optical tracking system that consists of a light-emitting camera and several light-reflecting optical markers attached to the head and the TMS coil for reference. This allows to save the correct coil location, orientation, and alignment to the real head, and also the digitization of the locations of the EEG electrodes. These features are vital for between session and subject reliability and for accurate stimulation of the dorsolateral prefrontal cortex. Once the stimulation target is identified using this system, it could be marked on a tape or a cap adjusted for each participant, and the stimulation site may be retained and recalled without the repeated use of this neuronavigation system.

Localization of DLPFC (will be computed offline before the TMS sessions). The left DLPFC will be identified using an MRI-guided neuronavigation system. The left-DLPFC target will be computed after the fMRI session, by evaluating the left-DLPFC surface location most adjacent to a voxels cluster that is the most anticorrelated with SGC activity during the rs-fMRI, and can produce maximal effective electric field, based on the head and coil models (Balderston et al., 2022; Cash et al., 2021).

Treatment with High Dose Theta-burst Stimulation () Procedures

Each participant's RMT will be determined. Treatment sessions: iTBS will be delivered at the individually targeted left-DLPFC site at 90% RMT. The stimulation pattern and total number of pulses will be triplet 50 Hz bursts, repeated at intervals of 200 ms (5 Hz); 2 s on and 8 s off; 1800 pulses per session; total duration of 10 min (Cole et al., 2021, 2020). There will be ten iTBS stimulation sessions/day on 5 sequential days. Stimulation will be double-blinded.

For both active and sham , we will use the Magventure Cool B65 A/P coil. The sham treatment works by blocking the magnetic field with an internal spacer on the sham side, allowing the operator to place the appropriate coil surface (active vs. sham) against the scalp. During the sham , we will use the coil's electric stimulation functionality that allows for the delivery of a brief electric pulse to the scalp simultaneous to the TMS pulse to mimic the scalp sensation during the sham condition. Importantly, the electric pulse is calibrated to the stimulator output to ensure a realistic sham condition.

Blinding Procedures: Patients who are enrolled in the study (i.e., met inclusion/exclusion criteria) will be randomized to active or Sham by a randomization system determined by the Independent Randomization Provider. This will occur at least one day before the start of treatment. Persons who will be blind to TMS status are patients and all study staff with the exception of Peter Chase (clinical coordinator at UCSD-IPC; and clinical and medical monitor, Dr. Michael Thase at UPenn-CNDS). TMS operator will be included in the blinding status.

Discontinuation Criteria

Discontinuation from the TMS will mean discontinuation from the study. Ending TMS due to AE will be

documented and patient will be evaluated for safety.
Participants are free to withdraw from participation in the study at any time upon request. The PI may discontinue or withdraw a participant from the study if deemed necessary for their health or safety.
We anticipate that some participants may withdraw. We do not expect early termination of participation due to patient or investigator withdrawal to have any impact on safety or well-being of participants. Patients who withdraw for AE will be evaluated by trained staff for safety.
Participants who enroll in the study (i.e., sign consent and pass screening) and are subsequently withdrawn will be replaced.

Data Management and Data Analysis
All data will be stored locally on the password protected computer used to administer the assessments and uploaded to a HIPAA compliant lab server that is managed by PI Hadas. Server data will separately store any participant identifying info (i.e. name, contact and date of birth) in a password protected file on the server that is only accessible with super-administrative privileges given to PIs, while all other computerized biomarker data will only be linked to a non-identifying alphanumeric participant ID that is accessible to authorized research staff personnel.
For purposes of analysis, authorized research study personnel will be able to download data from lab server in non-identifiable format only (i.e. data linked to participant study ID) onto the password protected lab computers. Data processing and statistical analyses will be conducted using MATLAB, Microsoft Excel, Python and SPSS software.
The clinical, MRI and neurophysiological data distributions, outliers, and baseline group differences will be assessed. Mixed models will assess significant group and condition differences for these outcome measures, at baseline and after treatment. The neurophysiological metrics will be correlated with the clinical assessments score change, and a classifier model will assess these metrics capabilities in predicting remission for each participant, looking at each arm.

Efficacy Assessments
The primary outcomes will be response to TMS as evidenced by change in MADRS and HRSD-17 scores for depression severity from the pre-treatment baseline to the end of the 5-day treatment course. The secondary outcome will be change TMS-EEG measures for cortical excitation, inhibitory and connectivity. Additional secondary measures will be changes for functional connectivity from pre-treatment to post-treatment using MRI of the brain.

10. HUMAN SUBJECTS

60 volunteers at UCSD-IPC and 34 volunteers at UPenn-CNDS, of any gender, 18-70 years of age who are diagnosed with BPD. All competent participants will provide written and informed consent. Potential participants will be invited to meet the research coordinator who will explain the rationale, procedures and potential risks as well as determine their eligibility for participation in the study.

Inclusion Criteria
In order to be eligible to participate in this study, an individual must meet all of the following criteria:

- Bipolar depression (BP I and BP II) by DSM 5 criteria (*Diagnostic and Statistical Manual of Mental Disorders*, 2013)
- Age 18-70
- Right or left handed
- All genders
- Treatment resistant depression, as in they must have treatment resistant depression with 2 or more prior antidepressant trials that have failed to produce a response (> 50% reduction in symptoms) using ATHF

criteria (Sackeim et al., 2019)

- Able to provide informed consent to participate in the study
- Must be on a stable medication regimen, requiring at least one mood stabilizer
- Depression severity as represented by scoring at least 20 on MADRS
- Meet the safety criteria as defined in the transcranial magnetic stimulation adult safety screen (TASS).

Exclusion Criteria

An individual who meets any of the following criteria will be excluded from participation in this study:

- No current substance abuse disorder for the past 6 months (previous substance abuse not exclusionary)
- Any psychotic disorder or current active psychotic symptoms (personality disorders not exclusionary unless in the opinion of the referring psychiatrist it would jeopardize participation)
- No dementia or other major neurological disorders
- Not having depression as primary disorder
- No major medical illness, for example metastatic cancer, end stage renal disease
- Not able to verify contact information. Participants must be able to follow through with the study & must have verified contact information and at least one verified contact
- Pregnancy. While there are no known risks to a fetus this is a new use of TMS, which has not been tested, thus pregnancy is exclusionary
- Score on YMRS greater than 12 (patients with mixed features have been shown not to respond well to TMS treatment (Tavares et al., 2021).
- Rapid cycling Bipolar illness (patients with > 4 mood episodes within the past year will be excluded, as they have a higher risk of switch to mania (Tondo et al., 2010)
- Any implants, conditions, or contraindications that would be deemed unsafe for TMS or MRI

Power Analysis

Previous HDS-TBS study for treatment resistant depression symptoms (open-labeled and without sham control) produced a clinical efficacy effect size larger than 1 (Cohen's-d) (Cole et al., 2021, 2020). When assigning a more conservative estimation of the effect-size to be 0.8 (Cohen's-d, two-tailed t-test, α=0.01, and a 1-β level of 0.80) the sufficient participants number found to be 39 for each group (or a total of 78 subjects). Given an attrition rate of about 15% we plan to include 60 participants at UCSD-IPC and 34 participants at UPenn-CNDS, to optimally power our clinical effects estimations. The neurophysiological effects (TMS-EEG at UCSD-IPC) and the fMRI effects (at UCSD-IPC and UPenn-CNDS), will be sufficiently powered with this group sizes.

11. RECRUITMENT AND PROCEDURES PREPARATORY TO RESEARCH

Individuals who are outpatients and are interested in treatment for BPD will be recruited from the UCSD Outpatient Psychiatry Clinics in 4S Ranch, La Jolla and Hillcrest, and the general San Diego community. Additional recruitment procedure will occur at UPenn-CNDS. Patients can express interest by initiating contact with the research. Patients who meet inclusion and exclusion criteria will be invited to the screening visit. Only IRB approved mailings and flyers materials will be utilized that describe the research opportunity, basic inclusion criteria, monetary compensation, and study contact information. We will also utilize IRB-approved on-line and radio ads for recruitment.

12. INFORMED CONSENT

Individuals who respond to the recruitment flyers and are eligible after the recruitment screening will be consented onsite at the study facilities, by phone, or via internet meeting. The consent form will be made available to all individuals as a paper copy will be mailed to them if they desire. Trained research study personnel, who

have completed online and lab-based training in HIPAA policy, human subjects research, and management of other research issues, will review the complete study procedures with the study participants as described in the consent forms. Research staff will ensure that all participant questions are answered. Study participants will sign the consent document. Only adults who provide signed consent will be considered enrolled subjects. Names and contact information will be collected for scheduling participants, and will also be maintained in password protected, encrypted files that are only accessible only to the treating clinicians and RA. These files will use an alphanumeric subject ID to link contact information with the separately stored password protected, encrypted and de-identified study data files; personal contact information will be destroyed after participation is complete.

Voluntary participation will be ensured by reading the following statement prior to the beginning of the study: 'You should understand that you are free to decide whether or not you want to participate in this study. If you do not want to participate, you are free to leave at any time and it will not affect any clinical treatment.'

If a participant is found ineligible at screening, or decides not to participate, all previously collected data will be destroyed immediately; no data will be retained.

13. ALTERNATIVES TO STUDY PARTICIPATION

This research may be registered as a clinical trial testing a clinically-prescribed treatment for depression with biomarker measurements. The alternative to participation is not to participate.

14. POTENTIAL RISKS

There are two main elements of the proposed treatment in this trial, which theoretically could pose a risk to participants, and involve firstly the treatment itself and secondly the study design. The inherent risks of the rTMS treatment are well documented: The most common, usually tolerable side effect is pain at the site of stimulation. There is also a theoretical risk of hearing loss: Earplugs will be used during rTMS sessions to prevent discomfort from the clicking noise generated by the stimulation and prevent hearing loss. No hearing loss has been found in humans exposed to TMS, despite extensive exposure to repeated stimulation over several years. Other side effects are discomfort in the surrounding muscles of stimulation, headache, and transient pre-syncope, as well as the rare adverse event of full syncope as well as very rare, but serious adverse event being seizure: safety guidelines were introduced more than 15 years ago (Rossi et al., 2021, 2009), and since their publication, the risk of seizures with rTMS is exceptionally low (1/10,000). Participation in this experimental study involves a blinded component in that the study participant is not aware of the experimental condition in each session. For patients who develop heightened suicide risk with clear safety issues during the study, we will offer immediate hospitalization and the option to leave the study and continue with known efficacious treatments such as electroconvulsive therapy. There will obviously be capacity for the blind to be broken in the case of a relevant medical need.

In terms of non-treatment elements of the study: MRI is not associated with any known health risks, and there is no proof that there will be short-term or long-term side effects. Exposure to magnetic stimulation or any strong magnetic field is not permitted in people who have a pacemaker, an implanted medication pump, a metal plate in the skull, or metal objects inside the eye or skull (e.g., after brain surgery or a shrapnel wound). Safety of TMS-EEG: EEG recordings are not associated with any known risks to health and there is no evidence that there are either short-term or long-term side effects though some scalp discomfort during the procedure which can occur in a small proportion of patients. Magnetic stimulators capitalize on the ability of time-varying magnetic fields to induce eddy currents in biologic tissue via the principle of electromagnetic induction. The magnetic stimulator stores electrical current and then discharges it in brief pulses through a stimulating coil. A magnetic field forms around the coil; with the magnetic flux lines perpendicular to the current flow. When the

resultant magnetic field is applied to a conducting medium, such as nervous tissue, an electrical current is induced in that medium that results in neuronal depolarization. This technique has been used to stimulate peripheral nerves and the central nervous system and has now become a tool for clinical neurophysiology. The ability of TMS to non-invasively stimulate brain areas presents a significant advance beyond techniques that require the invasive method of direct cortical or transcranial electrical stimulation. Magnetic fields pass through the scalp and skull without the impedance encountered by direct electrical stimulation, permitting enhanced control over the site and intensity of stimulation. In numerous studies, single-pulse TMS (<0.25 Hz and <0.5 Hz in paired-pulse) has been found to pose no significant health risk to properly screened healthy volunteers. Prospective studies designed to systematically evaluate health effects have not found changes in EEG, blood pressure, heart rate, serum cortisol, serum prolactin, cerebral blood flow, memory or cognition. Single-pulse TMS of the motor cortex has been used in children and infants as young as 2 weeks of age with no adverse effects reported. Single-pulse TMS is now in routine clinical diagnostic use in hundreds of neurophysiological laboratories worldwide. The induced electrical current is well below that which is expected to cause harm to nervous tissue. The US FDA has concluded that stimulation at <1 Hz carries virtually no seizure risk and is therefore classified as a non-significant risk device (Evans, 2007).

Questionnaires: The clinical, medical, and safety interviews and questionnaires are non-invasive clinical assessments associated with minimal risk. All measures are physically non-invasive, have been used in prior studies with seriously mentally ill participants, and have been found to elicit minimal distress or discomfort. They are not harmful or unpleasant. There may be discomfort and the possibility of frustration associated with the symptom ratings. Some participants may experience some embarrassment discussing personal information. There is the possibility that private or confidential information would be inadvertently disclosed, though the likelihood of this occurring is minimal. Data Management and Integrity Clinical Data Management: All clinical data will be collected on forms, which are identified by a unique participant ID number, participant initials, protocol ID number, and protocol phase and visit, and are entered into a secured REDCAP database which will be managed by the UCSD and UPenn REDCap (Research Electronic Data Capture) is a secure, web-based platform, developed by Vanderbilt University, which is used worldwide to collect all types of data ranging from research, to clinical, to operations. The system features a simple point-and-click interface for the design of data collection forms and surveys, custom query design functions for data export, extensive user rights management and logging for data security, mobile app integration, and many other useful modules which continue to be actively developed. Our site has considerable experience with clinical data management in REDCap. By adopting this platform for our research studies, we have greatly reduced project start-up time, simplified and streamlined our data collection strategy, and significantly increased the accuracy and integrity of the data we produce. A protocol data collection schedule will be used to monitor participant progress through the protocol, missing assessments, and other protocol deviations during the study. Data entry screens will incorporate range checks or lists of valid responses for each item. Forms with missing or invalid data in key identifying fields will be referred back to raters for correction before entry. Other missing or invalid data will not prevent the form from being entered but will be flagged for correction. Queries will be built around data entry and reports to review adverse events and side effects on a weekly basis, consistent with our current approach in federally-funded brain stimulation clinical trials. Participant confidentiality will be maintained by restricting study data access to specified study personnel. Most authorized personnel will have read-only access, and write/edit access will be restricted to study data management staff. Once data have been entered, an electronic audit trail will record all database changes. The database will reside on a central server, and all server data will be backed up on a regular basis. Outside access to the server will be restricted by a firewall. Regularly updated anti-virus software is installed on the server and all PCs linked to the server. Study participant identification numbers will be linked to treatment assignment in a separate, password-protected Access database file, with access restricted to the study biostatistician. During the study, this file will only be linked to the main database when required to generate interim reports relating adverse effects to treatment, or other interim analyses required by the Data and Safety Monitoring Board (DSMB), the Institutional Review Board (IRB), or any of the institutions of the

principal investigator or site principal investigators. At the conclusion of the study, originals of all data forms collected for this study will be archived and retained for the full period required by NIH regulations and relevant Californian laws governing the retention of medical records. Confidentiality and Privacy. All user access requires a preauthorized login name and password, and the system implements many security features such as extensive logging and automatic timeouts for idle logins. All data is organized under specific projects, and any user seeking access to data must be explicitly provided access to the project by the project's owner; typically the legally authorized Principal Investigator (PI). No sharing is enabled by default. Such explicit enabling of data access allows documentation and tracking of any data accesses and transfers. Data can be divided into multiple projects within a single multi-site research program. For example, with one project for each institutional scanning site, users, by default, have access only to the data in their institutional project. Once the data are uploaded, either the overall PI or their suitably authorized designates can share data into a separate common project, which is accessible to all of the authorized researchers within the program. If an outside researcher wishes to gain access to a subset of the data it can be provided through another shared project once authorization is obtained. In case data will be exported outside of UCSD or UPenn or presented, we will remove any protected health information (PHI).

15. RISK MANAGEMENT PROCEDURES AND ADEQUACY OF RESOURCES

Procedures for ensuring professional intervention. If a participant should become uncomfortable with any procedures, he/she can withdraw from the study at any time. If psychological distress, suicidal ideation, worsening mental health symptoms or another problem should occur during the study, the Co-Investigator (at UCSD: Zafiris J. Daskalakis, MD Ph.D., state-licensed psychiatrist; at UPenn: Michael Thase, MD) or a designated backup licensed professional will be contacted immediately to contact the participant and make recommendations. Additionally, we will take steps including (1) we provide to all participants an updated, ZIP-code customized list of local mental health programs, and (2) any disclosure of thoughts, intent, or plans to commit self-harm or suicide are immediately reported to the PI or his designee. If any emergency arises, 911 will be phoned immediately.

Electroencephalography (EEG). The procedures for EEG recording are considered safe and have been standardized for use in research for decades. The procedures are completely noninvasive and involve no physical or psychological harm. The tight fit of the EEG cap may cause temporary marks on the skin. Physical discomfort from wearing the device will be minimized by ensuring that it fits well.

Electrocardiography (EMG). The procedures for EMG recording are considered safe and have been standardized for use in research for decades. The procedures are completely noninvasive and involve no physical or psychological harm. Physical discomfort from the recording electrodes will be minimized.

Electrocardiography (ECG). The procedures for ECG recording are considered safe and have been standardized for use in research for decades. The procedures are completely noninvasive and involve no physical or psychological harm. Physical discomfort from wearing the device will be minimized by ensuring that it fits well.

Transcranial Magnetic Stimulation (TMS): We will follow two basic strategies to minimize risks associated with TMS: safe stimulation parameters and constant monitoring.

1) <u>Safe Stimulation parameters:</u> after motor threshold is determined, stimulation will be set at 90% of motor threshold and will be adjusted to scalp-brain distance. The stimulation intensity will not surpass 120% RMT according to clinical treatment guidelines (McClintock et al., 2017). The TMS-EEG single pulse protocol fires one pulse every 4 sec. or more, the paired-pulse protocol fires two pulses every 4 sec.

2) Monitoring: All participants will be monitored throughout the stimulation protocol by the TMS technicians and research associates (RA) trained at UCSD by Dr. Hadas, Dr. Daskalakis, Dr. Weissman and Dr. Appelbaum; or at UPenn by Dr. Michael Thase. The training will include (as per recs of Rossi et al., 2021), basic knowledge of brain physiology, basic mechanisms of TMS, the potential risks of the procedure and the physiological changes induced, as well as training around potential acute complications of TMS (recognition and management of seizures). At UCSD, Dr. Daskalakis or Dr. Weissman; or UPenn, Dr. Thase (or designated study physician) will be available on call for any other issues that arise**.** As per the consensus statement, stimulation parameters that follow these guidelines are considered "No risk" (Rossi et al., 2021).

The studies described here fall into the "Class 3" definition of clinical research (studies in normal subjects and patients that are expected to yield important data on brain physiology or on safety)(Rossi et al., 2009). Recommendations can be safely carried out by trained professionals, which include MDs, technicians, psychologists, physicists, physiologists, engineers, etc., under the responsibility of the clinical supervisor (At UCSD: Dr. Daskalakis, and Dr. Weissman ; At UPenn: Michael Thase, MD), whose physical presence in the lab is not required but who should be immediately available/on-call. In addition, a licensed physician (Dr. Daskalakis or designated study physician) will be the medically responsible clinician who will oversee screening procedures, assessment of risk factors, TMS parameters and application protocol and ensure subjects are adequately being monitored.

Management of Seizures. In the unlikely case of a TMS-induced seizure occurring, stimulation will be terminated immediately, subject safety will be ensured, and the RA will immediately notify the on-call clinical lead (At UCSD: Dr. Daskalakis, and Dr. Weissman ; At UPenn: Dr. Thase, or designated study physician) and PI (At UCSD: Dr. Hadas; At UPenn: Dr. Sheline). Emergent medical professionals (i.e., 911) will be called immediately. All seizures to date in published studies have been self-limited and occurred only during the treatment session, with no long-term adverse effects. There is no documented case of a TMS-related seizure resulting in status epilepticus or other life-threatening injury, so the basic precautions regarding seizure management will primarily involve stopping the stimulator, ensuring patient safety during the seizure and calling emergent medical personnel.

Confidentiality. Risks to privacy will be minimized by informing participants that all measurements and individual test results will be de-identified and treated confidentially. Written informed consent/assent and minimum identifying information collected from participants will have separate secure, electronic locations than securely stored study data so that so that individuals are not easily connected to the study results.

16. PRIVACY AND CONFIDENTIALITY CONSIDERATIONS INCLUDING DATA ACCESS AND MANAGEMENT

Participants will be informed about the limits of confidentiality, including that the information provided for this research study will not be shared with the exception of situations concerning safety (i.e., suicidal ideation or intent to harm self or others). No personal identifying information will be coded on the questionnaires, computerized test scores, or brain recording data. After electronically entering personal information (i.e., name, contact and date of birth), an alphanumeric participant ID number will automatically be assigned to each participant for all following assessments (i.e., cognitive behavioral data and neurophysiological data). Server data will store any participant identifying info (i.e., name, contact and date of birth) separately from all their study data in a password protected file on the server that is only accessible with superadmin privileges given to the PI, Dr. Hadas. Only the PI and study physician (UCSD: Dr. Weissman; UPenn: Dr. Thase) will have access to the file that links patient's health information (PHI) with the alphanumeric participant ID that is used for all clinical, imaging and neurophysiological data. All research study staff who accesses and manages the data will have completed on-line training in human subjects research, HIPAA, and clinical practices, lab training on research data management and confidentiality, and training to criterion on the project protocol. Data collected for research purposes, accessed

and managed by the trained research staff will be solely for the purposes of this research project. All data transfer of the de-identified study data onto the web server and then to lab computers will occur via secure encrypted connections. The lab computers storing study data will be password-protected and located behind secure and maintained firewalls and in locked office locations with authorized access.

17. POTENTIAL BENEFITS

A potential benefit from the for participants would be response or remission from their bipolar disorder or depression symptoms. There may or may not be a direct benefit to the participants. The information learned from this study will potentially benefit others with BPD in the future. Knowledge gained from this research could lead to better treatment of BPD. The findings from the neurophysiological biomarker investigations may help refine future treatments and help us understand the biology of this mental illness.

18. RISK/BENEFIT RATIO

The study protocol involves minimal risk for study participants and significant adverse events are not anticipated other than those discussed in the potential risks section. In the unlikely event that such events occur, serious and unanticipated and related adverse events will be reported in writing within 48 hours to the UCSD and UPenn IRB as well as any appropriate funding and regulatory agencies. The PI, Dr. Hadas and study physician (UCSD: Dr. Weissman; UPenn: Dr. Thase) will evaluate any adverse events and study data at regular intervals (monthly) and determine whether the adverse event affects the Risk/Benefit ratio of the study and whether modifications to the protocol (at Risks to Subjects) or assent/consent form (at Risks and Inconveniences) are required. During the review process, the principal investigator will evaluate whether the study should continue unchanged, require modification/amendment, continue or close to enrollment.

The study will inform how neuromodulation treatment can affect brain activity. We believe that our non-invasive procedures and the information gained from this study is important and the risk/benefit ratio appears favorable.

19. EXPENSE TO PARTICIPANT

The only anticipated expense to participants is the amount of time spent conducting the experiment.

20. COMPENSATION FOR PARTICIPATION

Every participant will receive $30 for each pre and post-treatment MRI session, $30 for each pre and post-treatment TMS-EEG session that may be conducted in combination with clinical assessment. Also $10 for each treatment day (5 treatment visit). The compensation for participating will be provided at the end of each visit, and will sum up to total of $170 by the end of the trial. All compensation will be given in the form of e-gift cards to retailers (e.g. Amazon). In compensation for travel expenses, participants may receive a total of $100.

21. PRIVILEGES/CERTIFICATIONS/LICENSES AND RESEARCH TEAM RESPONSIBILITIES

At UCSD:

Itay Hadas, Ph.D.: Principal Investigator. Dr. Hadas is a project scientist in the Department of Psychiatry at UCSD. Dr. Hadas has expertise in neurophysiology, cognitive neurosciences, specifically TMS-EEG methods. He is responsible for overseeing project activities for the TMS-EEG assessment aims, and will lead the analyses, preparation of manuscripts, and dissemination of results on these assessments.

Lawrence (Gregory) Appelbaum, Ph.D.: Co-Principal Investigator: Dr. Appelbaum is a Professor in the Department of Psychiatry at UCSD. Dr. Appelbaum has expertise in applied cognitive neurosciences. He is responsible for overseeing project activities for the TMS-EEG assessment aim, and will lead the analyses, preparation of manuscripts, and dissemination of results on these assessments.

Zafiris (Jeff) Daskalakis, M.D., Ph.D. – Co-Principal Investigator: Chair of the Department of Psychiatry at UCSD, Dr. Daskalakis is TMS privileged and leads the Interventional Psychiatry clinic. He will oversee TMS treatments and assist with the study procedures and their analytics as needed.

Cory Weissman, M.D. – Co-Principal Investigator: Dr. Weissman is an Assistant Professor in the Department of Psychiatry at UCSD. Dr. Weissman is a board-certified psychiatrist whose focus is providing care for patients with treatment-resistant mental illness. He has experience treating patients with severe mood, psychotic and substance use disorders in inpatient and outpatients settings. He will participate in analyses, preparation of manuscripts, and dissemination of results on these assessments.

Research Coordinator: will oversee the rTMS component of the trial working as a liaison between PI, the RA, and the rTMS technicians delivering the treatment.

TBN – Research Assistant: She/he will administer clinical assessments and TMS-EEG protocols. She/he will help coordinate assessment and treatment visits with the rTMS visits. The RA will also enter data.

At UPenn:

Yvette Sheline, MD

Claudia Baldassano, MD

Michael Thase, MD

Research Coordinator: will oversee the rTMS component of the trial working as a liaison between PI, the RA, and the rTMS technicians delivering the treatment.

22. BIBLIOGRAPHY

1. Addolorato, G., Antonelli, M., Cocciolillo, F., Vassallo, G.A., Tarli, C., Sestito, L., et al., 2017. Deep Transcranial Magnetic Stimulation of the Dorsolateral Prefrontal Cortex in Alcohol Use Disorder Patients: Effects on Dopamine Transporter Availability and Alcohol Intake. Eur. Neuropsychopharmacol. 27, 450–461. https://doi.org/10.1016/j.euroneuro.2017.03.008
2. Alyagon, U., Shahar, H., Hadar, A., Barnea-Ygael, N., Lazarovits, A., Shalev, H., et al., 2020. Alleviation of ADHD symptoms by non-invasive right prefrontal stimulation is correlated with EEG activity. NeuroImage Clin. 26, 102206. https://doi.org/10.1016/j.nicl.2020.102206
3. Balderston, N.L., Beer, J.C., Seok, D., Makhoul, W., Deng, Z.-D., Girelli, T., et al., 2022. Proof of concept study to develop a novel connectivity-based electric-field modelling approach for individualized targeting of transcranial magnetic stimulation treatment. Neuropsychopharmacology 47, 588–598. https://doi.org/10.1038/s41386-021-01110-6
4. Blumberger, D.M., Vila-Rodriguez, F., Thorpe, K.E., Feffer, K., Noda, Y., Giacobbe, P., et al., 2018. Effectiveness of theta burst versus high-frequency repetitive transcranial magnetic stimulation in patients with depression (THREE-D): a randomised non-inferiority trial. The Lancet 391, 1683–1692. https://doi.org/10.1016/s0140-6736(18)30295-2
5. Carmi, L., Alyagon, U., Barnea-Ygael, N., Zohar, J., Dar, R., Zangen, A., 2018. Clinical and electrophysiological outcomes of deep TMS over the medial prefrontal and anterior cingulate cortices in OCD patients. Brain Stimulat. 11, 158–165. https://doi.org/10.1016/j.brs.2017.09.004

6. Carvalho, A.F., Firth, J., Vieta, E., 2020. Bipolar Disorder. N. Engl. J. Med. 383, 58–66. https://doi.org/10.1056/NEJMra1906193
7. Cash, R.F.H., Cocchi, L., Lv, J., Wu, Y., Fitzgerald, P.B., Zalesky, A., 2021. Personalized connectivity-guided DLPFC-TMS for depression: Advancing computational feasibility, precision and reproducibility. Hum. Brain Mapp. https://doi.org/10.1002/hbm.25330
8. Cole, E.J., Phillips, A.L., Bentzley, B.S., Stimpson, K.H., Nejad, R., Barmak, F., et al., 2021. Stanford Neuromodulation Therapy (SNT): A Double-Blind Randomized Controlled Trial. Am. J. Psychiatry appi.ajp.2021.20101429. https://doi.org/10.1176/appi.ajp.2021.20101429
9. Cole, E.J., Stimpson, K.H., Bentzley, B.S., Gulser, M., Cherian, K., Tischler, C., et al., 2020. Stanford Accelerated Intelligent Neuromodulation Therapy for Treatment-Resistant Depression. Am. J. Psychiatry appi.ajp.2019.19070720. https://doi.org/10.1176/appi.ajp.2019.19070720
10. Diagnostic and Statistical Manual of Mental Disorders: Dsm-5, 2013. . American Psychiatric Pub Incorporated.
11. Dinur-Klein, L., Dannon, P., Hadar, A., Rosenberg, O., Roth, Y., Kotler, M., et al., 2014. Smoking Cessation Induced by Deep Repetitive Transcranial Magnetic Stimulation of the Prefrontal and Insular Cortices: A Prospective, Randomized Controlled Trial. Biol. Psychiatry, Alcoholism and Smoking 76, 742–749. https://doi.org/10.1016/j.biopsych.2014.05.020
12. Drevets, W.C., Price, J.L., Simpson Jr, J.R., Todd, R.D., Reich, T., Vannier, M., et al., 1997. Subgenual prefrontal cortex abnormalities in mood disorders. Nature 386, 824–827. https://doi.org/10.1038/386824a0
13. Drevets, W.C., Savitz, J., Trimble, M., 2008. The Subgenual Anterior Cingulate Cortex in Mood Disorders. CNS Spectr. 13, 663–681.
14. Evans, A., 2007. A review of the safety of transcranial magnetic stimulation: The Magstim Company Limited.
15. Farzan, F., Vernet, M., Shafi, M.M.D., Rotenberg, A., Daskalakis, Z.J., Pascual-Leone, A., 2016. Characterizing and Modulating Brain Circuitry through Transcranial Magnetic Stimulation Combined with Electroencephalography. Front. Neural Circuits 73. https://doi.org/10.3389/fncir.2016.00073
16. Fitzgerald, P.B., Benitez, J., de Castella, A., Daskalakis, Z.J., Brown, T.L., Kulkarni, J., 2006. A Randomized, Controlled Trial of Sequential Bilateral Repetitive Transcranial Magnetic Stimulation for Treatment-Resistant Depression. Am. J. Psychiatry 163, 88–94. https://doi.org/10.1176/appi.ajp.163.1.88
17. Fox, M.D., Buckner, R.L., White, M.P., Greicius, M.D., Pascual-Leone, A., 2012. Efficacy of Transcranial Magnetic Stimulation Targets for Depression Is Related to Intrinsic Functional Connectivity with the Subgenual Cingulate. Biol. Psychiatry, Novel Pharmacotherapies for Depression 72, 595–603. https://doi.org/10.1016/j.biopsych.2012.04.028
18. Hadas, I., Hadar, A., Lazarovits, A., Daskalakis, Z.J., Zangen, A., 2021. Right prefrontal activation predicts ADHD and its severity: A TMS-EEG study in young adults. Prog. Neuropsychopharmacol. Biol. Psychiatry 111, 110340. https://doi.org/10.1016/j.pnpbp.2021.110340
19. Hadas, I., Sun, Y., Lioumis, P., Zomorrodi, R., Jones, B., Voineskos, D., et al., 2019. Association of Repetitive Transcranial Magnetic Stimulation Treatment With Subgenual Cingulate Hyperactivity in Patients With Major Depressive Disorder: A Secondary Analysis of a Randomized Clinical Trial. JAMA Netw. Open 2, e195578–e195578. https://doi.org/10.1001/jamanetworkopen.2019.5578
20. Hajek, T., Gunde, E., Bernier, D., Slaney, C., Propper, L., Grof, P., et al., 2008. Subgenual cingulate volumes in affected and unaffected offspring of bipolar parents. J. Affect. Disord. 108, 263–269. https://doi.org/10.1016/j.jad.2007.10.024
21. Harrison, N.A., Brydon, L., Walker, C., Gray, M.A., Steptoe, A., Critchley, H.D., 2009. Inflammation Causes Mood Changes Through Alterations in Subgenual Cingulate Activity and Mesolimbic Connectivity. Biol. Psychiatry, Medical Consequences and Contributions to Depression 66, 407–414. https://doi.org/10.1016/j.biopsych.2009.03.015

22. Hill, A.T., Hadas, I., Zomorrodi, R., Voineskos, D., Fitzgerald, P.B., Blumberger, D.M., et al., 2021. Characterizing Cortical Oscillatory Responses in Major Depressive Disorder Before and After Convulsive Therapy: A TMS-EEG Study. J. Affect. Disord. 287, 78–88. https://doi.org/10.1016/j.jad.2021.03.010
23. Houenou, J., Wessa, M., Douaud, G., Leboyer, M., Chanraud, S., Perrin, M., et al., 2007. Increased white matter connectivity in euthymic bipolar patients: diffusion tensor tractography between the subgenual cingulate and the amygdalo-hippocampal complex. Mol. Psychiatry 12, 1001–1010. https://doi.org/10.1038/sj.mp.4002010
24. Kan, R.L.D., Zhang, B.B.B., Zhang, J.J.Q., Kranz, G.S., 2020. Non-invasive brain stimulation for posttraumatic stress disorder: a systematic review and meta-analysis. Transl. Psychiatry 10, 1–12. https://doi.org/10.1038/s41398-020-0851-5
25. Konstantinou, G., Hui, J., Ortiz, A., Kaster, T.S., Downar, J., Blumberger, D.M., et al., 2021. Repetitive transcranial magnetic stimulation (rTMS) in bipolar disorder: A systematic review. Bipolar Disord. https://doi.org/10.1111/bdi.13099
26. McClintock, S.M., Reti, I.M., Carpenter, L.L., McDonald, W.M., Dubin, M., Taylor, S.F., et al., 2017. Consensus Recommendations for the Clinical Application of Repetitive Transcranial Magnetic Stimulation (rTMS) in the Treatment of Depression. J. Clin. Psychiatry 78, 0–0. https://doi.org/10.4088/JCP.16cs10905
27. McGirr, A., Karmani, S., Arsappa, R., Berlim, M.T., Thirthalli, J., Muralidharan, K., et al., 2016. Clinical efficacy and safety of repetitive transcranial magnetic stimulation in acute bipolar depression. World Psychiatry 15, 85–86. https://doi.org/10.1002/wps.20300
28. Rossi, S., Antal, A., Bestmann, S., Bikson, M., Brewer, C., Brockmöller, J., et al., 2021. Safety and recommendations for TMS use in healthy subjects and patient populations, with updates on training, ethical and regulatory issues: Expert Guidelines. Clin. Neurophysiol. 132, 269–306. https://doi.org/10.1016/j.clinph.2020.10.003
29. Rossi, S., Hallett, M., Rossini, P.M., Pascual-Leone, A., 2009. Safety, ethical considerations, and application guidelines for the use of transcranial magnetic stimulation in clinical practice and research. Clin. Neurophysiol. 120, 2008–2039. https://doi.org/10.1016/j.clinph.2009.08.016
30. Rossini, P.M., Burke, D., Chen, R., Cohen, L.G., Daskalakis, Z., Di Iorio, R., et al., 2015. Non-invasive electrical and magnetic stimulation of the brain, spinal cord, roots and peripheral nerves: Basic principles and procedures for routine clinical and research application. An updated report from an I.F.C.N. Committee. Clin. Neurophysiol. 126, 1071–1107. https://doi.org/10.1016/j.clinph.2015.02.001
31. Sackeim, H.A., Aaronson, S.T., Bunker, M.T., Conway, C.R., Demitrack, M.A., George, M.S., et al., 2019. The assessment of resistance to antidepressant treatment: Rationale for the Antidepressant Treatment History Form: Short Form (ATHF-SF). J. Psychiatr. Res. 113, 125–136. https://doi.org/10.1016/j.jpsychires.2019.03.021
32. Sun, Y., Farzan, F., Mulsant, B.H., Rajji, T.K., Fitzgerald, P.B., Barr, M.S., et al., 2016. Indicators for Remission of Suicidal Ideation Following Magnetic Seizure Therapy in Patients With Treatment-Resistant Depression. JAMA Psychiatry 73, 337–345. https://doi.org/10.1001/jamapsychiatry.2015.3097
33. Tavares, D.F., Suen, P., Rodrigues dos Santos, C.G., Moreno, D.H., Lane Valiengo, L.D.C., Klein, I., et al., 2021. Treatment of mixed depression with theta-burst stimulation (TBS): results from a double-blind, randomized, sham-controlled clinical trial. Neuropsychopharmacology 46, 2257–2265. https://doi.org/10.1038/s41386-021-01080-9
34. Tischler, C., Gulser, M., Stimpson, K., Cole, E., Williams, N., 2019. Accelerated theta burst stimulation for Bipolar I and II: Assessing clinical changes pre- and post-treatment. Brain Stimul. Basic Transl. Clin. Res. Neuromodulation 12, 503. https://doi.org/10.1016/j.brs.2018.12.648
35. Tondo, L., Vázquez, G., Baldessarini, R.J., 2010. Mania associated with antidepressant treatment: comprehensive meta-analytic review. Acta Psychiatr. Scand. 121, 404–414.

https://doi.org/10.1111/j.1600-0447.2009.01514.x

23. FUNDING SUPPORT FOR THIS STUDY

This study will be supported through Development Proposal #36084: Bipolar efficacy biomarkers for accelerated intermittent theta burst rTMS trial.
Principal Investigator: Itay Hadas
Sponsor: THE MILKEN INSTITUTE
Sponsor Type: Private Non-Profit

This project will also get support from Dr. Z.J Daskalakis start-up funds.

24. BIOLOGICAL MATERIALS TRANSFER AGREEMENT

(N/A)

25. INVESTIGATIONAL DRUG FACT SHEET AND IND/IDE HOLDER

(N/A)

26. IMPACT ON STAFF

There will be no significant impact on clinical staff as a result of this study.

27. CONFLICT OF INTEREST

There are no conflicts of interest to report.

28. SUPPLEMENTAL INSTRUCTIONS FOR CANCER-RELATED STUDIES

(N/A)

29. OTHER APPROVALS/REGULATED MATERIALS

(N/A)

30. PROCEDURES FOR SURROGATE CONSENT AND/OR DECISIONAL CAPACITY ASSESSMENT

Surrogate consent will not be utilized for this study. We will not enroll those with impaired decision capacity.

UC San Diego
INSTITUTIONAL REVIEW BOARD ADMINISTRATION

Date: Friday, December 9th 2022

PI: Hadas, Itay

IRB# 800601, Version 37 in KIRB

Title: Bipolar efficacy biomarkers for accelerated intermittent theta burst rTMS trial

Type: Amendment

Review: Expedited by Non-Committee

Decision: Friday, December 9th 2022, valid through 11:59 pm (Pacific) Tuesday, March 28th 2023

The above review is complete with the following outcomes:

Your request to amend this project has been reviewed and approved using the expedited review process. The amendment included the following:

1. The Research Plan has been revised to add the Snaith-Hamilton Pleasure Scale (SHAPS) and Beck Depression Inventory (BDI) as assessments administered at screening, treatment visits 4-8, post-treatment visit 11 and open-label extension treatment visits 12-16. Additional administrations of the Young Mania Rating

Scale (YMRS) have also been added at baseline visit 3, treatment visits 4-8 and post-treatment visits 9 and 11. Additional clarifications have been made regarding the administration timepoints for other assessments.

The consents/assents were not revised. Re-consent is not required.

NOTE: IRB approval does not constitute other institutional required approvals. In the conduct of this research, the PI and study team must abide by UC San Diego PPM 100-5 (Responsibilities Section, Item D) and obtain any other approvals or permissions required by applicable laws or university policies.

If your study is a clinical trial you are reminded that applicable clinical trials must be registered on ClinicalTrials.gov. For more information or assistance, visit https://blink.ucsd.edu/sponsor/rci/clinical-trials.html or email the Research Compliance and Integrity Office at ctgov@ucsd.edu.

The following attachments are approved or acknowledged as part of this review:

Attachments

ICF_Bipolar v14_9.20.22_TRACKED.docx	Informed Consent/Parental Permission	Bipolar_ICF_v13_08.29.22_tracked
IRB_Milken_Bipolar_Research Plan_12.2.2022_TRACKED.docx	Protocol	Research Plan_Tracked_02DEC2022
aiTBS_Bipolar_flyer_Nov2021.docx	Recruitment Materials	Recruitment Flyer
EEG amplifier compumedics SynAmps2- FDA approval K023771.pdf	Investigator Brochure/Device Instructions	EEG (and ECG) Amplifier 510(k)
EMG amplifier Intronix Model 2024F story.pdf	Investigator Brochure/Device Instructions	EMG amplifier instructions
Etymotic ER1 tubephone insert Earphones.pdf	Investigator Brochure/Device Instructions	ear white noise ear phone instructions
MOP02-EN-03 - Magstim Bistim² Operating Manual.pdf	Investigator Brochure/Device Instructions	TMS stimulator (neurophysiological assessment)
Magventure Magpro 30 Family User Guide US-edition 5.0so 501-0965.pdf	Investigator Brochure/Device Instructions	rTMS (treatment stimulator)
Visor2 System User Guide 2.4.4.EN.pdf	Investigator Brochure/Device Instructions	MRI based TMS navigation system

UPenn CNDS - Sheline_850359_Study Protocol.docx	Other	UPenn site IRB protocol
UPenn CNDS - Sheline_850359_ICF.docx	Other	UPenn site - ICF form
ICF_Bipolar v14_9.20.22_CLEAN.pdf	Informed Consent/Parental Permission	Bipolar_ICF_v13_08.29.22_Clean
BIP_Participant Phone Screen and Script.pdf	Other	Phone Screener
IRB_Milken_Bipolar_Research Plan_12.2.2022_CLEAN.docx	Protocol	Research Plan Clean_02DEC2022

University of California San Diego · Office of IRB Administration

9500 Gilman Drive, Mail Code 0052 · La Jolla, CA 92093-0052

Tel: 858-246-4777 · E-mail: irb@health.ucsd.edu · Web: irb.ucsd.edu

UC San Diego
INSTITUTIONAL REVIEW BOARD ADMINISTRATION

Date: Wednesday, November 16th 2022

PI: Hadas, Itay

IRB# 800601, Version 35 in KIRB

Title: Bipolar efficacy biomarkers for accelerated intermittent theta burst rTMS trial

Type: Amendment

Review: Expedited by Non-Committee

Decision: Wednesday, November 16th 2022, valid through 11:59 pm (Pacific) Tuesday, March 28th 2023

The above review is complete with the following outcomes:

The amendment to add or remove study personnel has been approved.

Study personnel are individuals engaged in research activities involving living human individuals including accessing and/or using identifiable human specimens (e.g., blood, tissue) and/or identifiable private information for research purposes.

PI responsibilities include:
Study personnel have completed the appropriate CITI training.
Study personnel have been asked about potential conflicts of interest related to the research.
Study personnel engaged in research activities involving living human participants have the skills/training to perform their research-related responsibilities.

NOTE: IRB approval does not constitute other institutional required approvals. In the conduct of this research, the PI and study team must abide by UC San Diego PPM 100-5 (Responsibilities Section, Item D) and obtain any other approvals or permissions required by applicable laws or university policies.

If your study is a clinical trial you are reminded that applicable clinical trials must be registered on ClinicalTrials.gov. For more information or assistance, visit https://blink.ucsd.edu/sponsor/rci/clinical-trials.html or email the Research Compliance and Integrity Office at ctgov@ucsd.edu.

The following attachments are approved or acknowledged as part of this review:

Attachments

ICF_Bipolar v14_9.20.22_TRACKED.docx	Informed Consent/Parental Permission	Bipolar_ICF_v13_08.29.22_tracked
IRB_Milken_Bipolar_Research Plan_9.20.2022_TRACKED.docx	Protocol	Research Plan_Tracked_08.29.22
aiTBS_Bipolar_flyer_Nov2021.docx	Recruitment Materials	Recruitment Flyer
EEG amplifier compumedics SynAmps2- FDA approval K023771.pdf	Investigator Brochure/Device Instructions	EEG (and ECG) Amplifier 510(k)
EMG amplifier Intronix Model 2024F story.pdf	Investigator Brochure/Device Instructions	EMG amplifier instructions
Etymotic ER1 tubephone insert Earphones.pdf	Investigator Brochure/Device Instructions	ear white noise ear phone instructions
MOP02-EN-03 - Magstim Bistim² Operating Manual.pdf	Investigator Brochure/Device Instructions	TMS stimulator (neurophysiological assessment)
Magventure Magpro 30 Family User Guide US-edition 5.0so 501-0965.pdf	Investigator Brochure/Device Instructions	rTMS (treatment stimulator)
Visor2 System User Guide 2.4.4.EN.pdf	Investigator Brochure/Device	MRI based TMS navigation system

	Instructions	
UPenn CNDS - Sheline_850359_Study Protocol.docx	Other	UPenn site IRB protocol
UPenn CNDS - Sheline_850359_ICF.docx	Other	UPenn site - ICF form
ICF_Bipolar v14_9.20.22_CLEAN.pdf	Informed Consent/Parental Permission	Bipolar_ICF_v13_08.29.22_Clean
BIP_Participant Phone Screen and Script.pdf	Other	Phone Screener
IRB_Milken_Bipolar_Research Plan_9.20.2022_CLEAN.docx	Protocol	Clean protocol

University of California San Diego · Office of IRB Administration

9500 Gilman Drive, Mail Code 0052 · La Jolla, CA 92093-0052

Tel: 858-246-4777 · E-mail: irb@health.ucsd.edu · Web: irb.ucsd.edu

PROTOCOLS Kuali

UC San Diego
INSTITUTIONAL REVIEW BOARD ADMINISTRATION

Date: Monday, October 16th 2023

PI: Hadas, Itay

IRB# 800601, Version 69 in KIRB

Title: Bipolar efficacy biomarkers for accelerated intermittent theta burst rTMS trial

Type: Amendment

Review: Expedited by Non-Committee

Decision: Monday, October 16th 2023, valid through 11:59 pm (Pacific) Wednesday, March 27th 2024

The above review is complete with the following outcomes:

The amendment to add or remove study personnel has been approved.

Study personnel are individuals engaged in research activities involving living human individuals including accessing and/or using identifiable human specimens (e.g., blood, tissue) and/or identifiable private information for research purposes.

PI responsibilities include:
Study personnel have completed the appropriate CITI training.
Study personnel have been asked about potential conflicts of interest related to the research.
Study personnel engaged in research activities involving living human participants have the skills/training to perform their research-related responsibilities.

NOTE: IRB approval does not constitute other institutional required approvals. In the conduct of this research, the PI and study team must abide by UC San Diego PPM 100-5 (Responsibilities Section, Item D) and obtain any other approvals or permissions required by applicable laws or university policies.

If your study is a clinical trial you are reminded that applicable clinical trials must be registered on ClinicalTrials.gov. For more information or assistance, visit https://blink.ucsd.edu/sponsor/rci/clinical-trials.html or email the Research Compliance and Integrity Office at ctgov@ucsd.edu.

The following attachments are approved or acknowledged as part of this review:

Attachments

EEG amplifier compumedics SynAmps2- FDA approval K023771.pdf	FDA Documentation (To/From)	EEG (and ECG) Amplifier 510(k)
EMG amplifier Intronix Model 2024F story.pdf	Investigator Brochure/Device Instructions	EMG amplifier instructions
Etymotic ER1 tubephone insert Earphones.pdf	Investigator Brochure/Device Instructions	ear white noise ear phone instructions
MOP02-EN-03 - Magstim Bistim² Operating Manual.pdf	Investigator Brochure/Device Instructions	TMS stimulator (neurophysiological assessment)
Magventure Magpro 30 Family User Guide US-edition 5.0so 501-0965.pdf	Investigator Brochure/Device Instructions	rTMS (treatment stimulator)
Visor2 System User Guide 2.4.4.EN.pdf	Investigator Brochure/Device Instructions	MRI based TMS navigation system
UPenn CNDS - Sheline_850359_Study Protocol.docx	Other	UPenn site IRB protocol
UPenn CNDS - Sheline_850359_ICF.docx	Other	UPenn site - ICF form
CLEAN Bipolar ICF 082923.pdf	Informed Consent/Parental Permission	Bipolar_ICF_v14_08.29.23_Clean

BIP_Participant Phone Screen and Script.pdf	Recruitment Materials	Phone Screener
CLEAN Bipolar Protocol 082923.docx	Protocol	Research Plan Clean_29AUG2023
Rev. Bipolar Flyer 032123.pdf	Recruitment Materials	Recruitment Flyer
Rev-UCSD-Daskalakis- Bipolar Disorder Screening Form (4).pdf	Recruitment Materials	Build Clinical
BCFS00464-UCSD-Daskalakis-Bipolar Disorder- Ad Copy (2).pdf	Recruitment Materials	Build Clinical
BCFS00464-UCSD-Daskalakis-Bipolar-Disorder- Landing Page [updated].pdf	Recruitment Materials	Build Clinical
Flyer set 1.pdf	Recruitment Materials	05/23/23 V.1
Flyer Set 2.pdf	Recruitment Materials	5/23/23 V.1
Flyer Set 3.pdf	Recruitment Materials	5/23/23 V.1
Flyer set 4.pdf	Recruitment Materials	5/23/23 V.1
CLEAN_Provider Letter.pdf	Recruitment Materials	5/23/23 V.1
TRACKED Bipolar Protocol 082923.docx	Protocol	TRACKED Protocol 29AUG2023
TRACKED Bipolar ICF 082923.docx	Informed Consent/Parental Permission	TRACKED Informed Consent 29AUG2023
TRACKED_Provider Letter.pdf	Recruitment Materials	Provider Letter
BipolarFlyer800601(2).pdf	Recruitment Materials	Recruitment flyer

University of California San Diego · Office of IRB Administration

9500 Gilman Drive, Mail Code 0052 · La Jolla, CA 92093-0990

Tel: 858-246-4777 · E-mail: irb@health.ucsd.edu · Web: irb.ucsd.edu

UC San Diego
INSTITUTIONAL REVIEW BOARD ADMINISTRATION

Date: Friday, October 13th 2023

PI: Hadas, Itay

IRB# 800601, Version 67 in KIRB

Title: Bipolar efficacy biomarkers for accelerated intermittent theta burst rTMS trial

Type: Amendment

Review: Expedited by Non-Committee

Decision: Friday, October 13th 2023, valid through 11:59 pm (Pacific) Wednesday, March 27th 2024

The above review is complete with the following outcomes:

Your request to amend this project has been reviewed and approved using the expedited review process. The amendment included the following:

1. Addition of a New flyer to be distributed for recruitment purposes.
2. Included the revised letter that provides details about the TMS and ECT/MST trials conducted at the UCSD Interventional Psychiatry clinic to be sent to other providers for recruitment purposes. This letter only contains

minor modifications to include information about a new TMS trial (FREED) that is non-accelerated and currently recruiting.

The consents/assents were not revised. Re-consent is not required.

NOTE: IRB approval does not constitute other institutional required approvals. In the conduct of this research, the PI and study team must abide by UC San Diego PPM 100-5 (Responsibilities Section, Item D) and obtain any other approvals or permissions required by applicable laws or university policies.

If your study is a clinical trial you are reminded that applicable clinical trials must be registered on ClinicalTrials.gov. For more information or assistance, visit https://blink.ucsd.edu/sponsor/rci/clinical-trials.html or email the Research Compliance and Integrity Office at ctgov@ucsd.edu.

The following attachments are approved or acknowledged as part of this review:

Attachments

EEG amplifier compumedics SynAmps2- FDA approval K023771.pdf	FDA Documentation (To/From)	EEG (and ECG) Amplifier 510(k)
EMG amplifier Intronix Model 2024F story.pdf	Investigator Brochure/Device Instructions	EMG amplifier instructions
Etymotic ER1 tubephone insert Earphones.pdf	Investigator Brochure/Device Instructions	ear white noise ear phone instructions
MOP02-EN-03 - Magstim Bistim² Operating Manual.pdf	Investigator Brochure/Device Instructions	TMS stimulator (neurophysiological assessment)
Magventure Magpro 30 Family User Guide US-edition 5.0so 501-0965.pdf	Investigator Brochure/Device Instructions	rTMS (treatment stimulator)
Visor2 System User Guide 2.4.4.EN.pdf	Investigator Brochure/Device Instructions	MRI based TMS navigation system
UPenn CNDS - Sheline_850359_Study Protocol.docx	Other	UPenn site IRB protocol
UPenn CNDS - Sheline_850359_ICF.docx	Other	UPenn site - ICF form
CLEAN Bipolar ICF 082923.pdf	Informed Consent/Parental Permission	Bipolar_ICF_v14_08.29.23_Clean

BIP_Participant Phone Screen and Script.pdf	Recruitment Materials	Phone Screener
CLEAN Bipolar Protocol 082923.docx	Protocol	Research Plan Clean_29AUG2023
Rev. Bipolar Flyer 032123.pdf	Recruitment Materials	Recruitment Flyer
Rev-UCSD-Daskalakis- Bipolar Disorder Screening Form (4).pdf	Recruitment Materials	Build Clinical
BCFS00464-UCSD-Daskalakis-Bipolar Disorder- Ad Copy (2).pdf	Recruitment Materials	Build Clinical
BCFS00464-UCSD-Daskalakis-Bipolar-Disorder- Landing Page [updated].pdf	Recruitment Materials	Build Clinical
Flyer set 1.pdf	Recruitment Materials	05/23/23 V.1
Flyer Set 2.pdf	Recruitment Materials	5/23/23 V.1
Flyer Set 3.pdf	Recruitment Materials	5/23/23 V.1
Flyer set 4.pdf	Recruitment Materials	5/23/23 V.1
CLEAN_Provider Letter.pdf	Recruitment Materials	5/23/23 V.1
TRACKED Bipolar Protocol 082923.docx	Protocol	TRACKED Protocol 29AUG2023
TRACKED Bipolar ICF 082923.docx	Informed Consent/Parental Permission	TRACKED Informed Consent 29AUG2023
TRACKED_Provider Letter.pdf	Recruitment Materials	Provider Letter
BipolarFlyer800601(2).pdf	Recruitment Materials	Recruitment flyer

University of California San Diego · Office of IRB Administration

9500 Gilman Drive, Mail Code 0052 · La Jolla, CA 92093-0990

Tel: 858-246-4777 · E-mail: irb@health.ucsd.edu · Web: irb.ucsd.edu

UC San Diego
INSTITUTIONAL REVIEW
BOARD ADMINISTRATION

Date: Wednesday, September 21st 2022

PI: Hadas, Itay

IRB# 800601, Version 33 in KIRB

Title: Bipolar efficacy biomarkers for accelerated intermittent theta burst rTMS trial

Type: Amendment

Review: Full Board by IRB B (IRB00000353)

Decision: Wednesday, September 21st 2022, valid through 11:59 pm (Pacific) Wednesday, March 29th 2023

The above review is complete with the following outcomes:

Your request to amend this project was reviewed by the convened IRB on 8/18/2022 and 9/15/2022 with outcomes of modifications required to secure approval. With your modifications submitted 9/20/2022 and reviewed using the expedited review procedure, it has been approved. The amendment included the following:

1. The Research Plan and Informed Consent have been revised to now include a 5-day, open-label extension treatment for individuals who have been deemed non-responsive to the initial 5-day treatment regimen, defined

by a <50% change in MADRS score. Per standard procedures, the study blind cannot be broken until after the trial is closed to enrollment. Thus, all participants who are non-responders, whether they received active or placebo treatment during the initial phase, are eligible for open-label treatment. This supplemental extension treatment provides support for an exploratory aim to collect data on whether or not additional accelerated treatment translates to better efficacy in non-respondents. This has also been proposed in order to improve recruitment rates for potential participants who are hesitant about making the commitment with the potential of receiving placebo only.

Re-consent is not required. The Open-Label Extension period will only be offered to newly enrolled participants.

IRB approval of an amendment does not constitute other institutional required approvals of the given amendment. In the conduct of this research, the PI and study team must abide by UC San Diego PPM 100-5 (Responsibilities Section, Item D) and obtain any other approvals or permissions required by applicable laws or university policies.

NOTE: IRB approval does not constitute other institutional required approvals. In the conduct of this research, the PI and study team must abide by UC San Diego PPM 100-5 (Responsibilities Section, Item D) and obtain any other approvals or permissions required by applicable laws or university policies.

If your study is a clinical trial you are reminded that applicable clinical trials must be registered on ClinicalTrials.gov. For more information or assistance, visit https://blink.ucsd.edu/sponsor/rci/clinical-trials.html or email the Research Compliance and Integrity Office at ctgov@ucsd.edu.

The following attachments are approved or acknowledged as part of this review:

Attachments

ICF_Bipolar v14_9.20.22_TRACKED.docx	Informed Consent/Parental Permission	Bipolar_ICF_v13_08.29.22_tracked
IRB_Milken_Bipolar_Research Plan_9.20.2022_TRACKED.docx	Protocol	Research Plan_Tracked_08.29.22
aiTBS_Bipolar_flyer_Nov2021.docx	Recruitment Materials	Recruitment Flyer
EEG amplifier compumedics SynAmps2- FDA approval K023771.pdf	Investigator Brochure/Device Instructions	EEG (and ECG) Amplifier 510(k)
EMG amplifier Intronix Model 2024F story.pdf	Investigator Brochure/Device Instructions	EMG amplifier instructions
Etymotic ER1 tubephone insert Earphones.pdf	Investigator Brochure/Device	ear white noise ear phone instructions

	Instructions	
MOP02-EN-03 - Magstim Bistim² Operating Manual.pdf	Investigator Brochure/Device Instructions	TMS stimulator (neurophysiological assessment)
Magventure Magpro 30 Family User Guide US-edition 5.0so 501-0965.pdf	Investigator Brochure/Device Instructions	rTMS (treatment stimulator)
Visor2 System User Guide 2.4.4.EN.pdf	Investigator Brochure/Device Instructions	MRI based TMS navigation system
UPenn CNDS - Sheline_850359_Study Protocol.docx	Other	UPenn site IRB protocol
UPenn CNDS - Sheline_850359_ICF.docx	Other	UPenn site - ICF form
ICF_Bipolar v14_9.20.22_CLEAN.pdf	Informed Consent/Parental Permission	Bipolar_ICF_v13_08.29.22_Clean
BIP_Participant Phone Screen and Script.pdf	Other	Phone Screener
IRB_Milken_Bipolar_Research Plan_9.20.2022_CLEAN.docx	Protocol	Clean protocol

University of California San Diego · Office of IRB Administration

9500 Gilman Drive, Mail Code 0052 · La Jolla, CA 92093-0052

Tel: 858-246-4777 · E-mail: irb@health.ucsd.edu · Web: irb.ucsd.edu

UC San Diego
INSTITUTIONAL REVIEW BOARD ADMINISTRATION

Date: Tuesday, August 29th 2023

PI: Hadas, Itay

IRB# 800601, Version 64 in KIRB

Title: Bipolar efficacy biomarkers for accelerated intermittent theta burst rTMS trial

Type: Amendment

Review: Expedited by Non-Committee

Decision: Tuesday, August 29th 2023, valid through 11:59 pm (Pacific) Wednesday, March 27th 2024

The above review is complete with the following outcomes:

Your request to amend this project has been reviewed and approved using the expedited review process. The amendment included the following:

1. The Research Plan has been revised to clarify that certain fMRI scan sequences will be performed "if time permits." This was added due to the time restraints at the MRI center and to avoid protocol deviations resulting from not performing all identified scans. Similarly, to avoid other sources of protocol deviations, language

regarding stimulation delivery for the Pre-Treatment TMS-EEG now includes "approximately" when referencing the number of pulses to be delivered, “approximately” has been added with regard to TMS delivery duration and the amount of break time between sessions for the aiTBS treatment course, and “depending on participant tolerability" has been added with regard to stimulation intensity to allow for tolerability-based variation. The ICF has also been revised to indicate that between-session breaks will be approximately 50 minutes.
2. The Research Plan has been revised to reduce the approximate duration of the Pre-treatment TMS–EEG visit (Visit 3) from 4.5 to 3 hours, as reflected in the ICF.
3. The Research Plan has been revised to add that Active/Placebo Rating will occur after each participant’s first and fiftieth treatment sessions. The single question of whether or not the participant believes they are receiving active treatment or sham will be posed to reduce the risk of bias due to unblinding and prevent the overestimation of treatment benefits.
4. The ICF has been revised to remove the study-administered COVID-19 safety screening, as the clinic provides this to all patients as they check into the front desk.
5. The ICF has been revised to indicate that the post-study consult with the study psychiatrist is optional. It is noted that all enrolled participants have a primary psychiatrist of their own that they will have met with by this time, so requiring this of all participants represents an unnecessary burden.
6. Minor, clarifying and grammatical edits have been made throughout the Research Plan and ICF.
7. Caroline Huang and Joseph Liang have been added to the protocol as Key Persons.

Re-consent is not required. Changes do not represent new information that would reasonably impacts one's decision to continue participating.

NOTE: IRB approval does not constitute other institutional required approvals. In the conduct of this research, the PI and study team must abide by UC San Diego PPM 100-5 (Responsibilities Section, Item D) and obtain any other approvals or permissions required by applicable laws or university policies.

If your study is a clinical trial you are reminded that applicable clinical trials must be registered on ClinicalTrials.gov. For more information or assistance, visit https://blink.ucsd.edu/sponsor/rci/clinical-trials.html or email the Research Compliance and Integrity Office at ctgov@ucsd.edu.

The following attachments are approved or acknowledged as part of this review:

Attachments

EEG amplifier compumedics SynAmps2- FDA approval K023771.pdf	FDA Documentation (To/From)	EEG (and ECG) Amplifier 510(k)
EMG amplifier Intronix Model 2024F story.pdf	Investigator Brochure/Device Instructions	EMG amplifier instructions
Etymotic ER1 tubephone insert Earphones.pdf	Investigator Brochure/Device Instructions	ear white noise ear phone instructions
	Investigator	

MOP02-EN-03 - Magstim Bistim² Operating Manual.pdf	Brochure/Device Instructions	TMS stimulator (neurophysiological assessment)
Magventure Magpro 30 Family User Guide US-edition 5.0so 501-0965.pdf	Investigator Brochure/Device Instructions	rTMS (treatment stimulator)
Visor2 System User Guide 2.4.4.EN.pdf	Investigator Brochure/Device Instructions	MRI based TMS navigation system
UPenn CNDS - Sheline_850359_Study Protocol.docx	Other	UPenn site IRB protocol
UPenn CNDS - Sheline_850359_ICF.docx	Other	UPenn site - ICF form
CLEAN Bipolar ICF 082923.pdf	Informed Consent/Parental Permission	Bipolar_ICF_v14_08.29.23_Clean
BIP_Participant Phone Screen and Script.pdf	Recruitment Materials	Phone Screener
CLEAN Bipolar Protocol 082923.docx	Protocol	Research Plan Clean_29AUG2023
Rev. Bipolar Flyer 032123.pdf	Recruitment Materials	Recruitment Flyer
Rev-UCSD-Daskalakis- Bipolar Disorder Screening Form (4).pdf	Recruitment Materials	Build Clinical
BCFS00464-UCSD-Daskalakis-Bipolar Disorder- Ad Copy (2).pdf	Recruitment Materials	Build Clinical
BCFS00464-UCSD-Daskalakis-Bipolar-Disorder- Landing Page [updated].pdf	Recruitment Materials	Build Clinical
Flyer set 1.pdf	Recruitment Materials	05/23/23 V.1
Flyer Set 2.pdf	Recruitment Materials	5/23/23 V.1
Flyer Set 3.pdf	Recruitment Materials	5/23/23 V.1
Flyer set 4.pdf	Recruitment Materials	5/23/23 V.1
DVC_Merged_Recruitment_Letter.pdf	Recruitment Materials	5/23/23 V.1
TRACKED Bipolar Protocol 082923.docx	Protocol	TRACKED Protocol 29AUG2023
TRACKED Bipolar ICF 082923.docx	Informed Consent/Parental Permission	TRACKED Informed Consent 29AUG2023

University of California San Diego · Office of IRB Administration

9500 Gilman Drive, Mail Code 0052 · La Jolla, CA 92093-0990

Tel: 858-246-4777 · E-mail: irb@health.ucsd.edu · Web: irb.ucsd.edu

UC San Diego
INSTITUTIONAL REVIEW BOARD ADMINISTRATION

Date: Friday, July 29th 2022

PI: Hadas, Itay

IRB# 800601, Version 23 in KIRB

Title: Bipolar efficacy biomarkers for accelerated intermittent theta burst rTMS trial

Type: Amendment

Review: Expedited by Non-Committee

Decision: Friday, July 29th 2022, valid through 11:59 pm (Pacific) Wednesday, March 29th 2023

The above review is complete with the following outcomes:

Your request to amend this project has been reviewed and approved using the expedited review process. The amendment included the following:

1. The protocol has been revised to include procedures for obtaining remote consent. In situations where this applies, the consent will be emailed to the participants and a research member will go over it via phone, then obtain signature using UCSD DocuSign.

2. The protocol has been revised to indicate that eligibility will be determined prior consent via a phone screening process. A copy of the phone screen and script have been added.
3. The protocol has been revised to indicated that the Structured Clinical Interview for DSM Disorders (SCID) has been replaced with the Mini International Neuropsychiatric (MINI). The researchers feel that the MINI is less complex and will minimize participant discomfort compared to the SCID.
4. Drs. Michael Fitzgerald and Xia Li have been added to the protocol as Co-investigators

Re-consent is not required. No participants have yet enrolled.

IRB approval of an amendment does not constitute other institutional required approvals of the given amendment. In the conduct of this research, the PI and study team must abide by UC San Diego PPM 100-5 (Responsibilities Section, Item D) and obtain any other approvals or permissions required by applicable laws or university policies.

NOTE: IRB approval does not constitute other institutional required approvals. In the conduct of this research, the PI and study team must abide by UC San Diego PPM 100-5 (Responsibilities Section, Item D) and obtain any other approvals or permissions required by applicable laws or university policies.

If your study is a clinical trial you are reminded that applicable clinical trials must be registered on ClinicalTrials.gov. For more information or assistance, visit https://blink.ucsd.edu/sponsor/rci/clinical-trials.html or email the Research Compliance and Integrity Office at ctgov@ucsd.edu.

The following attachments are approved or acknowledged as part of this review:

Attachments

ICF_Bipolar v13_14JUL2022_tracked.docx	Informed Consent/Parental Permission	Bipolar_ICF_v13_14JUL2022_tracked
IRB_Milken_Bipolar_Research Plan_7.29.2022_tracked.docx	Protocol	Research Plan_Tracked_7.29.22
aiTBS_Bipolar_flyer_Nov2021.docx	Recruitment Materials	Recruitment Flyer
EEG amplifier compumedics SynAmps2-FDA approval K023771.pdf	Investigator Brochure/Device Instructions	EEG (and ECG) Amplifier 510(k)
EMG amplifier Intronix Model 2024F story.pdf	Investigator Brochure/Device Instructions	EMG amplifier instructions
Etymotic ER1 tubephone insert Earphones.pdf	Investigator Brochure/Device Instructions	ear white noise ear phone instructions
	Investigator	

MOP02-EN-03 - Magstim Bistim² Operating Manual.pdf	Brochure/Device Instructions	TMS stimulator (neurophysiological assessment)
Magventure Magpro 30 Family User Guide US-edition 5.0so 501-0965.pdf	Investigator Brochure/Device Instructions	rTMS (treatment stimulator)
Visor2 System User Guide 2.4.4.EN.pdf	Investigator Brochure/Device Instructions	MRI based TMS navigation system
UPenn CNDS - Sheline_850359_Study Protocol.docx	Other	UPenn site IRB protocol
UPenn CNDS - Sheline_850359_ICF.docx	Other	UPenn site - ICF form
ICF_Bipolar v13_14JUL2022_clean.pdf	Informed Consent/Parental Permission	Bipolar_ICF_v13_14JUL2022_Clean
IRB_Milken_Bipolar_Research Plan_7.29.2022_clean.docx	Protocol	Research Plan_Clean_7.29.22
BIP_Participant Phone Screen and Script.pdf	Other	Phone Screener

University of California San Diego · Office of IRB Administration

9500 Gilman Drive, Mail Code 0052 · La Jolla, CA 92093-0052

Tel: 858-246-4777 · E-mail: irb@health.ucsd.edu · Web: irb.ucsd.edu

UC San Diego
INSTITUTIONAL REVIEW BOARD ADMINISTRATION

Date: Friday, July 29th 2022

PI: Hadas, Itay

IRB# 800601, Version 23 in KIRB

Title: Bipolar efficacy biomarkers for accelerated intermittent theta burst rTMS trial

Type: Amendment

Review: Expedited by Non-Committee

Decision: Friday, July 29th 2022, valid through 11:59 pm (Pacific) Wednesday, March 29th 2023

The above review is complete with the following outcomes:

Your request to amend this project has been reviewed and approved using the expedited review process. The amendment included the following:

1. The protocol has been revised to include procedures for obtaining remote consent. In situations where this applies, the consent will be emailed to the participants and a research member will go over it via phone, then obtain signature using UCSD DocuSign.

2. The protocol has been revised to indicate that eligibility will be determined prior consent via a phone screening process. A copy of the phone screen and script have been added.
3. The protocol has been revised to indicated that the Structured Clinical Interview for DSM Disorders (SCID) has been replaced with the Mini International Neuropsychiatric (MINI). The researchers feel that the MINI is less complex and will minimize participant discomfort compared to the SCID.
4. Drs. Michael Fitzgerald and Xia Li have been added to the protocol as Co-investigators

Re-consent is not required. No participants have yet enrolled.

IRB approval of an amendment does not constitute other institutional required approvals of the given amendment. In the conduct of this research, the PI and study team must abide by UC San Diego PPM 100-5 (Responsibilities Section, Item D) and obtain any other approvals or permissions required by applicable laws or university policies.

NOTE: IRB approval does not constitute other institutional required approvals. In the conduct of this research, the PI and study team must abide by UC San Diego PPM 100-5 (Responsibilities Section, Item D) and obtain any other approvals or permissions required by applicable laws or university policies.

If your study is a clinical trial you are reminded that applicable clinical trials must be registered on ClinicalTrials.gov. For more information or assistance, visit https://blink.ucsd.edu/sponsor/rci/clinical-trials.html or email the Research Compliance and Integrity Office at ctgov@ucsd.edu.

The following attachments are approved or acknowledged as part of this review:

Attachments

ICF_Bipolar v13_14JUL2022_tracked.docx	Informed Consent/Parental Permission	Bipolar_ICF_v13_14JUL2022_tracked
IRB_Milken_Bipolar_Research Plan_7.29.2022_tracked.docx	Protocol	Research Plan_Tracked_7.29.22
aiTBS_Bipolar_flyer_Nov2021.docx	Recruitment Materials	Recruitment Flyer
EEG amplifier compumedics SynAmps2-FDA approval K023771.pdf	Investigator Brochure/Device Instructions	EEG (and ECG) Amplifier 510(k)
EMG amplifier Intronix Model 2024F story.pdf	Investigator Brochure/Device Instructions	EMG amplifier instructions
Etymotic ER1 tubephone insert Earphones.pdf	Investigator Brochure/Device Instructions	ear white noise ear phone instructions
	Investigator	

MOP02-EN-03 - Magstim Bistim² Operating Manual.pdf	Brochure/Device Instructions	TMS stimulator (neurophysiological assessment)
Magventure Magpro 30 Family User Guide US-edition 5.0so 501-0965.pdf	Investigator Brochure/Device Instructions	rTMS (treatment stimulator)
Visor2 System User Guide 2.4.4.EN.pdf	Investigator Brochure/Device Instructions	MRI based TMS navigation system
UPenn CNDS - Sheline_850359_Study Protocol.docx	Other	UPenn site IRB protocol
UPenn CNDS - Sheline_850359_ICF.docx	Other	UPenn site - ICF form
ICF_Bipolar v13_14JUL2022_clean.pdf	Informed Consent/Parental Permission	Bipolar_ICF_v13_14JUL2022_Clean
IRB_Milken_Bipolar_Research Plan_7.29.2022_clean.docx	Protocol	Research Plan_Clean_7.29.22
BIP_Participant Phone Screen and Script.pdf	Other	Phone Screener

University of California San Diego · Office of IRB Administration

9500 Gilman Drive, Mail Code 0052 · La Jolla, CA 92093-0052

Tel: 858-246-4777 · E-mail: irb@health.ucsd.edu · Web: irb.ucsd.edu

UC San Diego
INSTITUTIONAL REVIEW
BOARD ADMINISTRATION

Date: Monday, July 18th 2022

PI: Hadas, Itay

IRB# 800601, Version 18 in KIRB

Title: Bipolar efficacy biomarkers for accelerated intermittent theta burst rTMS trial

Type: Amendment

Review: Expedited by Non-Committee

Decision: Monday, July 18th 2022, valid through 11:59 pm (Pacific) Wednesday, March 29th 2023

The above review is complete with the following outcomes:

Your request to amend this project has been reviewed and approved using the expedited review process. The amendment included the following:

1. The Research Plan and Informed Consent have been revised throughout to replace the term HDS-TBS with aiTBS, as HDS is outdated.
2. The Research Plan and Informed Consent have been revised to more accurately reflect the anticipated time

commitments.
3. The scheduled activities tables in the Research Plan and Informed Consent have been revised to reflect that clinical assessments will not occur during visit 2 or visit 10, and that an fMRI scan will take place during visit 10. The UPenn Computerized Neurocognitive Battery (CNB) will no longer be administered.
4. Information regarding concomitant medications and recording of AE/SAEs has been added to the revised Research Plan.
5. The PHQ-9 and GAD-7 have been removed from the Research Plan, as they will no longer be administered.
6. The Informed Consent has been revised to remove language regarding remote visits, as all visits will now be in-person.
7. The reproductive risks section of the Informed Consent has been revised to remove "if you father a child" with "trying to conceive," as this new language is applicable to all participants.
8. Redundancies in the language regarding MRI have been removed from the Informed Consent.

Re-consent is not required. No participants have yet enrolled.

IRB approval of an amendment does not constitute other institutional required approvals of the given amendment. In the conduct of this research, the PI and study team must abide by UC San Diego PPM 100-5 (Responsibilities Section, Item D) and obtain any other approvals or permissions required by applicable laws or university policies.

The following attachments are approved or acknowledged as part of this review:

Attachments

ICF_Bipolar v13_14JUL2022_tracked.docx	Informed Consent/Parental Permission	Bipolar_ICF_v13_14JUL2022_tracked
IRB_Milken_Bipolar_Research Plan_7.13.2022_tracked.docx	Protocol	Research Plan_Tracked_7.13.22
aiTBS_Bipolar_flyer_Nov2021.docx	Recruitment Materials	Recruitment Flyer
EEG amplifier compumedics SynAmps2-FDA approval K023771.pdf	Investigator Brochure/Device Instructions	EEG (and ECG) Amplifier 510(k)
EMG amplifier Intronix Model 2024F story.pdf	Investigator Brochure/Device Instructions	EMG amplifier instructions
Etymotic ER1 tubephone insert Earphones.pdf	Investigator Brochure/Device Instructions	ear white noise ear phone instructions
MOP02-EN-03 - Magstim Bistim² Operating Manual.pdf	Investigator Brochure/Device Instructions	TMS stimulator (neurophysiological assessment)
Magventure Magpro 30 Family User Guide	Investigator	

US-edition 5.0so 501-0965.pdf	Brochure/Device Instructions	rTMS (treatment stimulator)
Visor2 System User Guide 2.4.4.EN.pdf	Investigator Brochure/Device Instructions	MRI based TMS navigation system
UPenn CNDS - Sheline_850359_Study Protocol.docx	Other	UPenn site IRB protocol
UPenn CNDS - Sheline_850359_ICF.docx	Other	UPenn site - ICF form
ICF_Bipolar v13_14JUL2022_clean.pdf	Informed Consent/Parental Permission	Bipolar_ICF_v13_14JUL2022_Clean
IRB_Milken_Bipolar_Research Plan_7.13.2022_clean.pdf	Protocol	Research Plan_Clean_7.13.22

University of California San Diego · Office of IRB Administration

9500 Gilman Drive, Mail Code 0052 · La Jolla, CA 92093-0052

Tel: 858-246-4777 · E-mail: irb@health.ucsd.edu · Web: irb.ucsd.edu

UC San Diego
INSTITUTIONAL REVIEW BOARD ADMINISTRATION

Date: Friday, July 1st 2022

PI: Hadas, Itay

IRB# 800601, Version 15 in KIRB

Title: Bipolar efficacy biomarkers for accelerated intermittent theta burst rTMS trial

Type: Amendment

Review: Expedited by Non-Committee

Decision: Friday, July 1st 2022, valid through 11:59 pm (Pacific) Wednesday, March 29th 2023

The above review is complete with the following outcomes:

Your request to amend this project has been reviewed and approved using the expedited review process. The amendment included the following:

1. A new post-treatment follow-up visit has been added to the protocol and described in the revised Informed Consent. This visit includes administration of clinical assessments and should take about 45 minutes to complete. Participants will be compensated $15 for this visit.

2. Alexander Wen, Molly Malone, and Deepinder Nagra have been added to the study as Key Persons.

Re-consent is not required. No participants have yet been enrolled.

IRB approval of an amendment does not constitute other institutional required approvals of the given amendment. In the conduct of this research, the PI and study team must abide by UC San Diego PPM 100-5 (Responsibilities Section, Item D) and obtain any other approvals or permissions required by applicable laws or university policies.

The following attachments are approved or acknowledged as part of this review:

Attachments

ICF_Bipolar v10_11MAY2022_TRACKED.docx	Informed Consent/Parental Permission	ICF_Tracked_11MAY2022
IRB_Milken_Bipolar_Research Plan_6.13.2022_TRACKED.docx	Protocol	Research Plan_Tracked_6.13.22
aiTBS_Bipolar_flyer_Nov2021.docx	Recruitment Materials	Recruitment Flyer
EEG amplifier compumedics SynAmps2- FDA approval K023771.pdf	Investigator Brochure/Device Instructions	EEG (and ECG) Amplifier 510(k)
EMG amplifier Intronix Model 2024F story.pdf	Investigator Brochure/Device Instructions	EMG amplifier instructions
Etymotic ER1 tubephone insert Earphones.pdf	Investigator Brochure/Device Instructions	ear white noise ear phone instructions
MOP02-EN-03 - Magstim Bistim² Operating Manual.pdf	Investigator Brochure/Device Instructions	TMS stimulator (neurophysiological assessment)
Magventure Magpro 30 Family User Guide US-edition 5.0so 501-0965.pdf	Investigator Brochure/Device Instructions	rTMS (treatment stimulator)
Visor2 System User Guide 2.4.4.EN.pdf	Investigator Brochure/Device Instructions	MRI based TMS navigation system
UPenn CNDS - Sheline_850359_Study Protocol.docx	Other	UPenn site IRB protocol
UPenn CNDS - Sheline_850359_ICF.docx	Other	UPenn site - ICF form
ICF_Bipolar v11_CLEAN.pdf	Informed Consent/Parental	ICF_Bipolar v11_Clean_

UC San Diego
INSTITUTIONAL REVIEW
BOARD ADMINISTRATION

Date: Thursday, June 8th 2023

PI: Hadas, Itay

IRB# 800601, Version 58 in KIRB

Title: Bipolar efficacy biomarkers for accelerated intermittent theta burst rTMS trial

Type: Amendment

Review: Expedited by Non-Committee

Decision: Thursday, June 8th 2023, valid through 11:59 pm (Pacific) Wednesday, March 27th 2024

The above review is complete with the following outcomes:

The amendment to update study personnel has been approved.

Study personnel are individuals engaged in research activities involving living human individuals including accessing and/or using identifiable human specimens (e.g., blood, tissue) and/or identifiable private information for research purposes.

PI responsibilities include:
Study personnel have completed the appropriate CITI training.
Study personnel have been asked about potential conflicts of interest related to the research.
Study personnel engaged in research activities involving living human participants have the skills/training to perform their research-related responsibilities.

NOTE: IRB approval does not constitute other institutional required approvals. In the conduct of this research, the PI and study team must abide by UC San Diego PPM 100-5 (Responsibilities Section, Item D) and obtain any other approvals or permissions required by applicable laws or university policies.

If your study is a clinical trial you are reminded that applicable clinical trials must be registered on ClinicalTrials.gov. For more information or assistance, visit https://blink.ucsd.edu/sponsor/rci/clinical-trials.html or email the Research Compliance and Integrity Office at ctgov@ucsd.edu.

The following attachments are approved or acknowledged as part of this review:

Attachments

EEG amplifier compumedics SynAmps2- FDA approval K023771.pdf	FDA Documentation (To/From)	EEG (and ECG) Amplifier 510(k)
EMG amplifier Intronix Model 2024F story.pdf	Investigator Brochure/Device Instructions	EMG amplifier instructions
Etymotic ER1 tubephone insert Earphones.pdf	Investigator Brochure/Device Instructions	ear white noise ear phone instructions
MOP02-EN-03 - Magstim Bistim² Operating Manual.pdf	Investigator Brochure/Device Instructions	TMS stimulator (neurophysiological assessment)
Magventure Magpro 30 Family User Guide US-edition 5.0so 501-0965.pdf	Investigator Brochure/Device Instructions	rTMS (treatment stimulator)
Visor2 System User Guide 2.4.4.EN.pdf	Investigator Brochure/Device Instructions	MRI based TMS navigation system
UPenn CNDS - Sheline_850359_Study Protocol.docx	Other	UPenn site IRB protocol
UPenn CNDS - Sheline_850359_ICF.docx	Other	UPenn site - ICF form
ICF_Bipolar v14_9.20.22_CLEAN.pdf	Informed Consent/Parental Permission	Bipolar_ICF_v13_08.29.22_Clean

BIP_Participant Phone Screen and Script.pdf	Recruitment Materials	Phone Screener
CLEAN Bipolar Protocol_15MAR2023.docx	Protocol	Research Plan Clean_02DEC2022
Rev. Bipolar Flyer 032123.pdf	Recruitment Materials	Recruitment Flyer
Rev-UCSD-Daskalakis- Bipolar Disorder Screening Form (4).pdf	Recruitment Materials	Build Clinical
BCFS00464-UCSD-Daskalakis-Bipolar Disorder- Ad Copy (2).pdf	Recruitment Materials	Build Clinical
BCFS00464-UCSD-Daskalakis-Bipolar-Disorder- Landing Page [updated].pdf	Recruitment Materials	Build Clinical
Flyer set 1.pdf	Recruitment Materials	05/23/23 V.1
Flyer Set 2.pdf	Recruitment Materials	5/23/23 V.1
Flyer Set 3.pdf	Recruitment Materials	5/23/23 V.1
Flyer set 4.pdf	Recruitment Materials	5/23/23 V.1
DVC_Merged_Recruitment_Letter.pdf	Recruitment Materials	5/23/23 V.1

University of California San Diego · Office of IRB Administration

9500 Gilman Drive, Mail Code 0052 · La Jolla, CA 92093-0990

Tel: 858-246-4777 · E-mail: irb@health.ucsd.edu · Web: irb.ucsd.edu

PROTOCOLS Kuali

UC San Diego
INSTITUTIONAL REVIEW BOARD ADMINISTRATION

Date: Tuesday, May 23rd 2023

PI: Hadas, Itay

IRB# 800601, Version 56 in KIRB

Title: Bipolar efficacy biomarkers for accelerated intermittent theta burst rTMS trial

Type: Amendment

Review: Expedited by Non-Committee

Decision: Tuesday, May 23rd 2023, valid through 11:59 pm (Pacific) Wednesday, March 27th 2024

The above review is complete with the following outcomes:

Your request to amend this project has been reviewed and approved using the expedited review process. The amendment included the following changes in Study Procedures:

-The PI Partnered with Build Clinical- Benji Hochberger, Founder & CEO, 914-954-1816, a digital health advertising technology company to accelerate recruitment efforts. Includes the following eight new recruitment documents.

Flyer 1
Flyer 2
Flyer 3
Flyer 4
BCFS00464 UCSD Daskalakis Bipolar Disorder Landing page
BCFS00464 UCSD Daskalakis Bipolar Disorder AD copy
BCFS00464 UCSD Daskalakis Bipolar Disorder Screening form
REV Bipolar Flyer
-A “DVC Merged Recruitment letter” has been added to mail out to local providers to increase recruitment efforts across all our trials.

The consents/assents were not revised. Re-consent is not required.

NOTE: IRB approval does not constitute other institutional required approvals. In the conduct of this research, the PI and study team must abide by UC San Diego PPM 100-5 (Responsibilities Section, Item D) and obtain any other approvals or permissions required by applicable laws or university policies.

If your study is a clinical trial you are reminded that applicable clinical trials must be registered on ClinicalTrials.gov. For more information or assistance, visit https://blink.ucsd.edu/sponsor/rci/clinical-trials.html or email the Research Compliance and Integrity Office at ctgov@ucsd.edu.

The following attachments are approved or acknowledged as part of this review:

Attachments

EEG amplifier compumedics SynAmps2- FDA approval K023771.pdf	FDA Documentation (To/From)	EEG (and ECG) Amplifier 510(k)
EMG amplifier Intronix Model 2024F story.pdf	Investigator Brochure/Device Instructions	EMG amplifier instructions
Etymotic ER1 tubephone insert Earphones.pdf	Investigator Brochure/Device Instructions	ear white noise ear phone instructions
MOP02-EN-03 - Magstim Bistim² Operating Manual.pdf	Investigator Brochure/Device Instructions	TMS stimulator (neurophysiological assessment)
Magventure Magpro 30 Family User Guide US-edition 5.0so 501-0965.pdf	Investigator Brochure/Device Instructions	rTMS (treatment stimulator)
Visor2 System User Guide 2.4.4.EN.pdf	Investigator Brochure/Device Instructions	MRI based TMS navigation system

UPenn CNDS - Sheline_850359_Study Protocol.docx	Other	UPenn site IRB protocol
UPenn CNDS - Sheline_850359_ICF.docx	Other	UPenn site - ICF form
ICF_Bipolar v14_9.20.22_CLEAN.pdf	Informed Consent/Parental Permission	Bipolar_ICF_v13_08.29.22_Clean
BIP_Participant Phone Screen and Script.pdf	Recruitment Materials	Phone Screener
CLEAN Bipolar Protocol_15MAR2023.docx	Protocol	Research Plan Clean_02DEC2022
Rev. Bipolar Flyer 032123.pdf	Recruitment Materials	Recruitment Flyer
Rev-UCSD-Daskalakis- Bipolar Disorder Screening Form (4).pdf	Recruitment Materials	Build Clinical
BCFS00464-UCSD-Daskalakis-Bipolar Disorder- Ad Copy (2).pdf	Recruitment Materials	Build Clinical
BCFS00464-UCSD-Daskalakis-Bipolar-Disorder- Landing Page [updated].pdf	Recruitment Materials	Build Clinical
Flyer set 1.pdf	Recruitment Materials	05/23/23 V.1
Flyer Set 2.pdf	Recruitment Materials	5/23/23 V.1
Flyer Set 3.pdf	Recruitment Materials	5/23/23 V.1
Flyer set 4.pdf	Recruitment Materials	5/23/23 V.1
DVC_Merged_Recruitment_Letter.pdf	Recruitment Materials	5/23/23 V.1

University of California San Diego · Office of IRB Administration

9500 Gilman Drive, Mail Code 0052 · La Jolla, CA 92093-0990

Tel: 858-246-4777 · E-mail: irb@health.ucsd.edu · Web: irb.ucsd.edu

UC San Diego
INSTITUTIONAL REVIEW BOARD ADMINISTRATION

Date: Wednesday, May 11th 2022

PI: Hadas, Itay

IRB# 800601, Version 12 in KIRB

Title: Bipolar efficacy biomarkers for accelerated intermittent theta burst rTMS trial

Type: Amendment

Review: Expedited by the admin

Decision: Wednesday, May 11th 2022, valid through 11:59 pm (Pacific) Wednesday, March 29th 2023

The above review is complete with the following outcomes:

Your request to amend this project has been reviewed and approved using the expedited review process. The amendment included the following:

1. Addition of new cognitive measurements: NIH Toolbox Emotion and Cognition Battery and the UPenn CNB (Computerized Neurocognitive Battery). These clinical assessments take approximately 20 minutes to complete.
2. Clarification that the Structured Clinical Interview for DSM-5 (SCID) will be used for this study, which the protocol

states. However, there were typos in the research plan and the Mini International Neuropsychiatric Interview (MINI).

Re-consent is not required. No participants have yet been enrolled.

IRB approval of an amendment does not constitute other institutional required approvals of the given amendment. In the conduct of this research, the PI and study team must abide by UC San Diego PPM 100-5 (Responsibilities Section, Item D) and obtain any other approvals or permissions required by applicable laws or university policies.

The following attachments are approved or acknowledged as part of this review:

Attachments

ICF_Bipolar v10_11MAY2022_TRACKED.docx	Informed Consent/Parental Permission	ICF_Tracked_11MAY2022
IRB_Milken_Bipolar_Research Plan_5.9.2022_TRACKED.docx	Protocol	Research Plan_Tracked_5.9.22
aiTBS_Bipolar_flyer_Nov2021.docx	Recruitment Materials	Recruitment Flyer
EEG amplifier compumedics SynAmps2- FDA approval K023771.pdf	Investigator Brochure/Device Instructions	EEG (and ECG) Amplifier 510(k)
EMG amplifier Intronix Model 2024F story.pdf	Investigator Brochure/Device Instructions	EMG amplifier instructions
Etymotic ER1 tubephone insert Earphones.pdf	Investigator Brochure/Device Instructions	ear white noise ear phone instructions
MOP02-EN-03 - Magstim Bistim² Operating Manual.pdf	Investigator Brochure/Device Instructions	TMS stimulator (neurophysiological assessment)
Magventure Magpro 30 Family User Guide US-edition 5.0so 501-0965.pdf	Investigator Brochure/Device Instructions	rTMS (treatment stimulator)
Visor2 System User Guide 2.4.4.EN.pdf	Investigator Brochure/Device Instructions	MRI based TMS navigation system
UPenn CNDS - Sheline_850359_Study Protocol.docx	Other	UPenn site IRB protocol
UPenn CNDS - Sheline_850359_ICF.docx	Other	UPenn site - ICF form
ICF_Bipolar v10_11MAY2022_CLEAN.pdf	Informed Consent/Parental Permission	ICF_Clean_1 1MAY2022

University of California San Diego · Office of IRB Administration

9500 Gilman Drive, Mail Code 0052 · La Jolla, CA 92093-0052

Tel: 858-246-4777 · E-mail: irb@health.ucsd.edu · Web: irb.ucsd.edu

UC San Diego
INSTITUTIONAL REVIEW BOARD ADMINISTRATION

Date: Tuesday, April 25th 2023

PI: Hadas, Itay

IRB# 800601, Version 52 in KIRB

Title: Bipolar efficacy biomarkers for accelerated intermittent theta burst rTMS trial

Type: Amendment

Review: Expedited by Non-Committee

Decision: Tuesday, April 25th 2023, valid through 11:59 pm (Pacific) Wednesday, March 27th 2024

The above review is complete with the following outcomes:

The amendment to add or remove study personnel has been approved.

Study personnel are individuals engaged in research activities involving living human individuals including accessing and/or using identifiable human specimens (e.g., blood, tissue) and/or identifiable private information for research purposes.

PI responsibilities include:
Study personnel have completed the appropriate CITI training.
Study personnel have been asked about potential conflicts of interest related to the research.
Study personnel engaged in research activities involving living human participants have the skills/training to perform their research-related responsibilities.

NOTE: IRB approval does not constitute other institutional required approvals. In the conduct of this research, the PI and study team must abide by UC San Diego PPM 100-5 (Responsibilities Section, Item D) and obtain any other approvals or permissions required by applicable laws or university policies.

If your study is a clinical trial you are reminded that applicable clinical trials must be registered on ClinicalTrials.gov. For more information or assistance, visit https://blink.ucsd.edu/sponsor/rci/clinical-trials.html or email the Research Compliance and Integrity Office at ctgov@ucsd.edu.

The following attachments are approved or acknowledged as part of this review:

Attachments

ICF_Bipolar v14_9.20.22_TRACKED.docx	Informed Consent/Parental Permission	Bipolar_ICF_v13_08.29.22_tracked
TRACKED Bipolar Protocol_21MAR2023.docx	Protocol	Research Plan_Tracked_02DEC2022
aiTBS_Bipolar_flyer_Nov2021.docx	Recruitment Materials	Recruitment Flyer
EEG amplifier compumedics SynAmps2- FDA approval K023771.pdf	Investigator Brochure/Device Instructions	EEG (and ECG) Amplifier 510(k)
EMG amplifier Intronix Model 2024F story.pdf	Investigator Brochure/Device Instructions	EMG amplifier instructions
Etymotic ER1 tubephone insert Earphones.pdf	Investigator Brochure/Device Instructions	ear white noise ear phone instructions
MOP02-EN-03 - Magstim Bistim² Operating Manual.pdf	Investigator Brochure/Device Instructions	TMS stimulator (neurophysiological assessment)
Magventure Magpro 30 Family User Guide US-edition 5.0so 501-0965.pdf	Investigator Brochure/Device Instructions	rTMS (treatment stimulator)
Visor2 System User Guide 2.4.4.EN.pdf	Investigator Brochure/Device	MRI based TMS navigation system

	Instructions	
UPenn CNDS - Sheline_850359_Study Protocol.docx	Other	UPenn site IRB protocol
UPenn CNDS - Sheline_850359_ICF.docx	Other	UPenn site - ICF form
ICF_Bipolar v14_9.20.22_CLEAN.pdf	Informed Consent/Parental Permission	Bipolar_ICF_v13_08.29.22_Clean
BIP_Participant Phone Screen and Script.pdf	Other	Phone Screener
CLEAN Bipolar Protocol_15MAR2023.docx	Protocol	Research Plan Clean_02DEC2022
Rev. Bipolar Flyer 032123.pdf	Recruitment Materials	Recruitment Flyer

University of California San Diego · Office of IRB Administration

9500 Gilman Drive, Mail Code 0052 · La Jolla, CA 92093-0990

Tel: 858-246-4777 · E-mail: irb@health.ucsd.edu · Web: irb.ucsd.edu

UC San Diego
INSTITUTIONAL REVIEW BOARD ADMINISTRATION

Date: Friday, April 8th 2022

PI: Hadas, Itay

IRB# 800601, Version 8 in KIRB

Title: Bipolar efficacy biomarkers for accelerated intermittent theta burst rTMS trial

Type: Amendment

Review: Expedited by Non-Committee

Decision: Friday, April 8th 2022, valid through 11:59 pm (Pacific) Wednesday, March 29th 2023

The above review is complete with the following outcomes:

Your request to amend this project has been reviewed and approved on an expedited basis. The amendment included the following:
1. Updated the Key Personnel List
IRB approval of an amendment does not constitute other institutional required approvals of the given amendment. In the conduct of this research, the PI and study team must abide by UC San Diego PPM 100-5 (Responsibilities Section, Item Your request to amend this project has been reviewed and acknowledged.

The following attachments are approved or acknowledged as part of this review:

Attachments

ICF_aiTBS_Bipolar ver3.0_new_revised_final_rule.docx	Informed Consent/Parental Permission	Informed Consent Form track changes
IRB_Milken_Bipolar_IRB_protocol_application_Feb_11.docx	Protocol	Study Protocol
aiTBS_Bipolar_flyer_Nov2021.docx	Recruitment Materials	Recruitment Flyer
EEG amplifier compumedics SynAmps2- FDA approval K023771.pdf	Investigator Brochure/Device Instructions	EEG (and ECG) Amplifier 510(k)
EMG amplifier Intronix Model 2024F story.pdf	Investigator Brochure/Device Instructions	EMG amplifier instructions
Etymotic ER1 tubephone insert Earphones.pdf	Investigator Brochure/Device Instructions	ear white noise ear phone instructions
MOP02-EN-03 - Magstim Bistim² Operating Manual.pdf	Investigator Brochure/Device Instructions	TMS stimulator (neurophysiological assessment)
Magventure Magpro 30 Family User Guide US-edition 5.0so 501-0965.pdf	Investigator Brochure/Device Instructions	rTMS (treatment stimulator)
Visor2 System User Guide 2.4.4.EN.pdf	Investigator Brochure/Device Instructions	MRI based TMS navigation system
UPenn CNDS - Sheline_850359_Study Protocol.docx	Other	UPenn site IRB protocol
UPenn CNDS - Sheline_850359_ICF.docx	Other	UPenn site - ICF form
ICF_aiTBS_Bipolar ver3.0_new_revised_final_rule.pdf	Informed Consent/Parental Permission	ICF clean version

University of California San Diego · Office of IRB Administration

9500 Gilman Drive, Mail Code 0052 · La Jolla, CA 92093-005

Tel: 858-246-4777 · E-mail: irb@health.ucsd.edu · Web: irb.ucsd.edu

UC San Diego
INSTITUTIONAL REVIEW BOARD ADMINISTRATION

Date: Wednesday, March 30th 2022

PI: Hadas, Itay

IRB# 800601, Version 6 in KIRB

Title: Bipolar efficacy biomarkers for accelerated intermittent theta burst rTMS trial

Type: Initial

Review: Full Board by IRB B (IRB00000353)

Decision: Wednesday, March 30th 2022, valid through 11:59 pm (Pacific) Wednesday, March 29th 2023

The above review is complete with the following outcomes:

The above-referenced project was reviewed and approved by one of this institution's Institutional Review Boards in accordance with the requirements of the Code of Federal Regulations on the Protection of Human Subjects (45 CFR 46 and 21 CFR 50 and 56), including its relevant Subparts.

The IRB has determined the investigational device associated with this study is a Non-Significant Risk Device in that it does not meet the criteria for a Significant Risk Device per the criteria outlined in 21 CFR 812.3(m) including that the device does not present a potential for serious risk to the health, safety, or welfare of a subject. The study must follow all the abbreviated regulations at 21 CFR 812.2(b).

The IRB determined that this project presents no more than minimal risk to human subjects in that the probability and magnitude of harm or discomfort anticipated in the research are not greater in and of themselves than those ordinarily encountered in daily life or during the performance of routine physical or psychological examinations or tests.

The protocol listed the following funding (or potential funding) information:
Sponsor: The Milken Institute

In the conduct of this research, the PI and study team must abide by UC San Diego PPM 100-5 (Responsibilities Section, Item D) and obtain any other approvals or permissions required by applicable laws or university policies.

The following attachments are approved or acknowledged as part of this review:

Attachments

ICF_aiTBS_Bipolar ver3.0_new_revised_final_rule.docx	Informed Consent/Parental Permission	Informed Consent Form track changes
IRB_Milken_Bipolar_IRB_protocol_application_Feb_11.docx	Protocol	Study Protocol
aiTBS_Bipolar_flyer_Nov2021.docx	Recruitment Materials	Recruitment Flyer
EEG amplifier compumedics SynAmps2- FDA approval K023771.pdf	Investigator Brochure/Device Instructions	EEG (and ECG) Amplifier 510(k)
EMG amplifier Intronix Model 2024F story.pdf	Investigator Brochure/Device Instructions	EMG amplifier instructions
Etymotic ER1 tubephone insert Earphones.pdf	Investigator Brochure/Device Instructions	ear white noise ear phone instructions
MOP02-EN-03 - Magstim Bistim² Operating Manual.pdf	Investigator Brochure/Device Instructions	TMS stimulator (neurophysiological assessment)
Magventure Magpro 30 Family User Guide US-edition 5.0so 501-0965.pdf	Investigator Brochure/Device Instructions	rTMS (treatment stimulator)
Visor2 System User Guide 2.4.4.EN.pdf	Investigator Brochure/Device Instructions	MRI based TMS navigation system
UPenn CNDS - Sheline_850359_Study Protocol.docx	Other	UPenn site IRB protocol
UPenn CNDS - Sheline_850359_ICF.docx	Other	UPenn site - ICF form
