# Supplementary material for: Accelerated Intermittent Theta-Burst Stimulation for Treatment-Resistant Bipolar Depression: A Randomized Clinical Trial
Source: JAMA Netw Open. 2025 Feb 11;8(2):e2459361. doi: 10.1001/jamanetworkopen.2024.59361 (PMC11815521; doi:10.1001/jamanetworkopen.2024.59361)
Supplement: Supplement 2. — eMethods. eFigure. CONSORT Flow Chart eTable 1. Inclusion/Exclusion Criteria eTable 2. Target x, y, and z Coordinates and Coil Orientation Used for Treatment eTable 3. Guesses, Confidence, and Actual Treatment Assignments eReferences. [file jamanetwopen-e2459361-s002.pdf]

Supplemental Online Content

Appelbaum LG, Daniels H, Lochhead L, et al. Accelerated intermittent theta-burst stimulation for treatment-resistant bipolar depression: a randomized clinical trial. *JAMA Netw Open*. 2025;8(2):e2459361. doi:10.1001/jamanetworkopen.2024.59361

eMethods.
eFigure. CONSORT Flow Chart
eTable 1. Inclusion/Exclusion Criteria
eTable 2. Target x, y, and z Coordinates and Coil Orientation Used for Treatment
eTable 3. Guesses, Confidence, and Actual Treatment Assignments
eReferences.

This supplemental material has been provided by the authors to give readers additional information about their work.

© 2025 Appelbaum LG et al. *JAMA Network Open*.

eMethods

1.1. Overview:

This pre-registered (NCT05393648) double-blind, randomized, sham-controlled trial followed CONSORT reporting guidelines and was approved by the UCSD institutional review board. The full protocol lasted approximately 13 weeks and involved screening and enrollment, clinical assessment, baseline neuroimaging and neurophysiology that was used to compute personalized and optimized left dorsolateral prefrontal cortex (DLPFC) targets, five consecutive days of hourly active or sham stimulation sessions, and post-treatment re-measurement of clinical, neuroimaging and neurophysiology assessments. All study activities occurred at the University of California, San Diego within the Interventional Psychiatry Program and associated imaging facilities between the dates of July 21, 2022 and March 8, 2024.

The following sections detail the research methods and participant characteristics leading to analysis of clinical efficacy using the Montgomery-Åsberg Depression Rating Scale (MADRS; [1]) study primary endpoint. The primary objective of this study was to compare change in MADRS scores, from pre-treatment to post-treatment, with the hypothesis that significantly greater symptom reduction would be observed following active stimulation versus sham stimulation. Study design and implementation parallel those in Sheline et al. [2], with differences described below.

1.2. Participants:

Patients diagnosed with Bipolar I or Bipolar II Disorder, between 18 and 70 years of age in a current depressive episode were eligible for this study. Participants were required to have treatment-resistant depression (i.e., having undergone two or more prior antidepressant trials that have failed to produce a response, as defined by the Anti-Depressant Treatment History Form; [3]), and a score greater than 19 on the MADRS at screening. Patients with active psychotic symptoms, scores higher than 12 on the Young Mania Rating Scale (YMRS), or a history of seizures were excluded. All participants provided written informed consent.

© 2025 Appelbaum LG et al. *JAMA Network Open*.

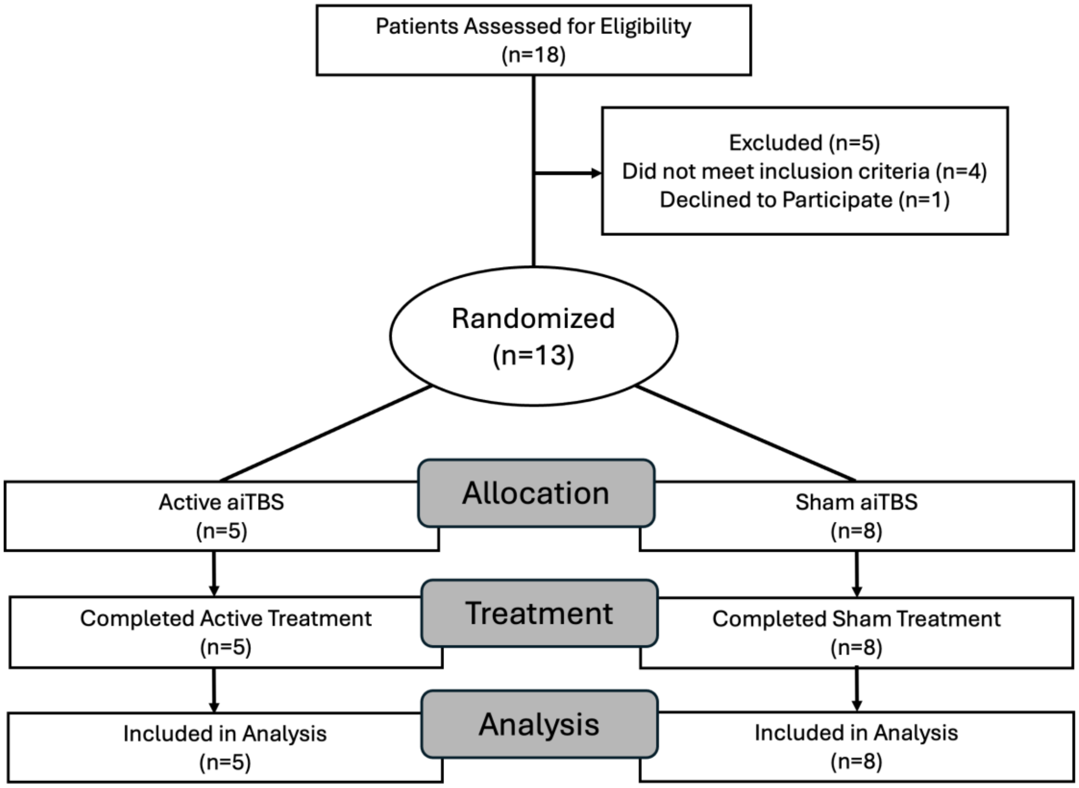

***eFigure**. CONSORT flow chart illustrating the screening, allocation, follow-up, and analysis participant information*

Inclusion	Exclusion
• Bipolar depression (BP I and BP II) by DSM 5 criteria (*Diagnostic and Statistical Manual of Mental Disorders*, 2013).<br>• Age 18-70.<br>• Right or left-handed.<br>• All genders.<br>• Treatment resistant depression, as in they must have treatment resistant depression with 2 or more prior antidepressant trials that have failed to produce a response (> 50% reduction in symptoms) using ATHF criteria [3].<br>• Able to provide informed consent to participate in the study.<br>• Must be on a stable medication regimen, requiring at least one mood stabilizer.	• Current substance abuse disorder for the past 6 months (previous substance abuse not exclusionary).<br>• Any psychotic disorder or current active psychotic symptoms (personality disorders not exclusionary unless in the opinion of the referring psychiatrist it would jeopardize participation).<br>• Dementia or other major neurological disorders<br>• Not having depression as primary disorder.<br>• Major medical illness, for example metastatic cancer or end stage renal disease.<br>• Not able to verify contact information. Participants must be able to follow through with the study & must have verified contact information and at least one verified contact.

© 2025 Appelbaum LG et al. *JAMA Network Open*.

• Depression severity as represented by scoring at least 20 on MADRS.<br>• Mini-Mental State Examination (MMSE)>24.<br>• Meet the safety criteria as defined in the transcranial magnetic stimulation adult safety screen (TASS).	• Pregnancy. While there are no known risks to a fetus this is a new use of TMS, which has not been tested, thus pregnancy is exclusionary.<br>• Score on YMRS greater than 12 (patients with mixed features have been shown not to respond well to TMS treatment [4]).<br>• Rapid cycling Bipolar illness (patients with > 4 mood episodes within the past year will be excluded, as they have a higher risk of switch to mania [5]).<br>• Any implants, conditions, or contraindications that would be deemed unsafe for TMS or MRI.<br>• >40% change on MADRS between screening and start of treatment.

***eTable 1**. Inclusion/Exclusion Criteria*

Based on the methods of Hedeker and colleagues [6] a planned group size of 12 patients per arm was anticipated in order to provide 80% power for a large effect (*d* = 1.0) for a group by time interaction, assuming a correlation of 0.5 between the repeated measures. As illustrated in **eFigure 1**, eighteen participants were recruited into the study, with four not meeting inclusion criteria and one declining to participate. The remaining thirteen participants met inclusion/exclusion criteria, listed in **eTable 1**, and were randomized into the study with five patients allocated to the active treatment arm and eight allocated to the sham arm. Treatment assignment was not equal because randomization was done listwise and the full planned allotment was not achieved because the period of funding ended, leading to an unbalanced sample.

All five of the participants allocated to the active treatment completed the full five-day treatment protocol, as well as both the 1-week and 4-week follow up visits. Six of the eight participants allocated to the sham treatment arm completed the full five-day treatment protocol, as well as both the 1-week and 4-week follow up visits, while one participant completed all treatments but missed the fifth treatment day assessment, and a separate patient missed the four-week follow up visit.

At the completion of the trial, nine participants qualified for a free open-label extension (OLE) by exhibiting a <50% reduction in MADRS scores between pre-treatment baseline and 1-week post. Eight of these individuals enrolled and completed the OLE.

1.3. Demographics

A total of 13 patients were randomized into the sham treatment group (n=8) and the active treatment group (n=5). The sham group had a mean age of 41.75 years (SD = 19.3), while the active group had a mean age of 49.8 years (SD = 10.1). In the sham group, four participants were female and four were male, compared to four females and one male in the active group. In the sham group, one participant

© 2025 Appelbaum LG et al. *JAMA Network Open*.

was African American or Black, three were Hispanic or Latino, and four were White. In the active group, no participants were African American or Black, two were Hispanic or Latino, and three were White. The average educational attainment was similar between the two groups, with the sham group averaging 15.7 years (SD = 1.8) and the active group averaging 15.8 years (SD = 2.3).

1.4. Clinical Assessments

The primary clinical measure for this study was the Montgomery-Asberg Depression Rating Scale (MADRS [1]). This scale was administered before treatment, and at the 1- and 4-week post treatment follow ups with questions asked in relation to symptoms experienced in the past seven days. Pre-treatment MADRS assessments were collected at screening (19 days before treatment on average) and again at the neurophysiology visit (5.25 days before treatment on average) to assure that symptoms had not substantially changed. The MADRS was also collected at approximately the 5th treatment session of each daily visit, with the questions asked pertaining to symptoms over the preceding 24-hours. Notably the post-treatment MADRS scales reported in Sheline et al., [2] were collected at 1-day and 4-weeks following treatment, differing from the current study where they were collected at 1- and 4-weeks. In order to align the baseline symptom scales, the current analysis used the screening MARDS scores, as was done in [2].

In addition to the MADRS assessments, the Young Mania Rating Scale (YMRS), The Beck Scale for Suicide Ideation (SSI), and the 17-item Hamilton Rating Scale for Depression (HRSD-17) were completed pre-treatment and at the 1-week post visit. These assessments were also collected daily during the treatment week and at the 4-week post visit, along with the Snaith-Hamilton Pleasure Scale (SHAPS) and the Beck's Depression Inventory (BDI). The NIH Cognitive Toolbox was also administered at the pre- and post-treatment imaging visits.

1.5. Diagnoses and Medications

Three participants in the sham group and two participants in the active group were diagnosed with BDII, while three participants in the sham group and two participants in the active group had BDI. One participant in the sham group and one participant in the active group had a diagnosis of Bipolar disorder, unspecified. The sham group had an average of five prior antidepressant treatment trials (SD = 1.8), while the active group averaged 5.6 trials (SD = 1.5). Participants in the sham group had an average of 0.6 augmentation trials (SD = 0.8), compared to one trial (SD = 0.7) in the active group.

At the time of screening, three participants in the sham group and three participants in the active group had a history of treatment with Lithium. Additionally, seven participants in the sham group and three participants in the active group had a history of anticonvulsant treatment. In terms of antidepressants, four participants in the sham group had been treated with SSRIs, compared to four participants in the active group. SNRI treatment was recorded for three participants in the sham group and three participants in the active group. Four participants in the sham group had a history of treatment with other antidepressants, compared to three participants in the active group.

© 2025 Appelbaum LG et al. *JAMA Network Open*.

Throughout the duration of the trial, all participants maintained a stable medication regimen that included at least one mood stabilizer. In the sham group, one participant was taking lithium, compared to two participants in the active group. Five participants in the sham group and four participants in the active group were on anticonvulsants. Regarding antidepressants, three participants in the sham group and one participant in the active group were taking SSRIs. SNRI treatment was recorded for one participant in the sham group and one participant in the active group. For other antidepressants, three participants in the sham group and two participants in the active group were receiving treatment. Three participants in the sham group and four participants in the active group were on atypical antipsychotics.

The most common psychiatric comorbidity in the sham group was Generalized Anxiety Disorder, present in seven participants, compared to three participants in the active group. Additionally, one participant in the sham group had an eating disorder, compared to one participant in the active group. ADHD was diagnosed in two participants in the sham group.

Two participants in the sham group had hypothyroidism, compared to one participant in the active group. Migraines were present in four participants in the sham group and one participant in the active group. Hypertension was diagnosed in one participant in the sham group and one participant in the active group. Furthermore, one participant in the sham group had multiple sclerosis, and two participants in the sham group had a GERD diagnosis.

1.6. Neuroimaging and Target Generation:

Following screening and enrollment, twelve of the thirteen participants underwent a neuroimaging session consisting of anatomical and resting-state functional connectivity (RSFC) fMRI scans. RSFC was used to compute personalized left dorsolateral prefrontal cortex (DLPFC) targets by connectivity to the subgenual anterior cingulate cortex using seed map and cluster methodology as detailed elsewhere by Cash and colleagues [7]. Stimulation targeting was further optimized using electric-field modeling to account for differences in neuroanatomy using the SimNIBS software and either the methods developed by Balderson and colleagues [8] or the Targeting and Analysis Pipeline (TAP; [9]). The fourteenth participant was unable to complete the MRI scan and therefore published MNI coordinates [10] were used to derive their stimulation target (**eTable 2**), which was delivered at fixed at a 135-degree orientation.

1.6.1. Imaging Data Collection:

Scanning was conducted using a Siemens 3T Magnetom Prisma Fit scanner with a 64-channel head coil (Siemens, Erlangen, Germany) at the University of California, San Diego. Each session included the acquisition of 20 minutes of BOLD resting-state fMRI data (gradient-echo EPI, 2 mm isotropic resolution, 52° flip angle, TR/TE = 37/800 ms) along with field maps for distortion correction. These were collected in four individual, 5-minute scans, two with anterior-posterior orientations and two with posterior-anterior orientations. Participants were instructed to focus on a designated point on the wall outside the scanner, which they viewed through an overhead mirror attached to the head coil. T1- and

© 2025 Appelbaum LG et al. *JAMA Network Open*.

T2*-weighted structural images were also acquired for functional volume registration. The T1-weighted MPRAGE sequence had 0.8 mm isotropic resolution, an 8° flip angle, and TR/TE of 2400/2.22 ms. The T2*-weighted SPACE sequence had 0.8 mm isotropic resolution, a variable flip angle, and TR/TE of 3200/563 ms.

1.6.2. fMRI Analysis:

MRI images were preprocessed using fMRIPrep version 20.0.62 with ICA-AROMA, followed by xcpEngine version 1.1.03 using the fc-36p_despike pipeline. Anatomical brain images were extracted, segmented, and normalized to the Montreal Neurological Institute (MNI) standard space. BOLD reference images were motion-corrected through six-parameter rigid body realignment and co-registered to corresponding T1-weighted images using affine transformation with the boundary-based registration cost function. Susceptibility distortion correction was applied using pepolar field maps, and the BOLD timeseries were resampled to MNI standard space. Additional preprocessing included despiking, confound regression, and bandpass filtering with a 0.01–0.08 Hz passband. Voxelwise homogeneity was calculated, and spatial smoothing was applied using a 6 mm FWHM SUSAN kernel. Global signal regression was employed to account for respiration artifacts.

The individualized stimulation target in the left dlPFC for each participant was identified based on their baseline resting-state scans. The target location was determined using the cluster-based approach described by Cash and colleagues [7], which identifies regions within a dlPFC mask that exhibit the strongest negative correlation with the sgACC. The sgACC signal was defined using the seed map methodology, and a subset of the most negatively correlated, spatially clustered voxels was selected. The center of gravity of the largest voxel cluster was then designated as the stimulation target coordinate.

1.7. Electric-field Modeling:

Electric-field (e-field) modeling was used to improve and optimize the target by personalizing the coil orientation and maximizing the e-field amplitude at the stimulation site for each patient. This process accounted for changes in electrical conductivity between cerebrospinal fluid, gray matter, and white matter, as well as variations in the subject's dlPFC gyral shape and orientation, which influence current distribution depending on coil positioning. Finite element models of the skin, skull, gray matter, white matter, and CSF were generated from T1 and T2 scans using SimNIBS version 2.1. The stimulation site, identified from the connectivity data, was then optimized using one of two closely-related modeling approaches developed by Balderson and colleagues [8] and Dannhauer and colleagues [9], with six patients receiving optimization using each algorithm. In each approach, E-field distributions were sampled at the stimulation site, and the coil orientation that produced the highest e-field at that location was selected for the stimulation sessions. Individual patient target coordinates and coil orientations are presented in **eTable 2.**

© 2025 Appelbaum LG et al. *JAMA Network Open*.

Participant ID	x	y	z	Coil Orientation	E-field Method
002	-38.1	40.4	52.4	39.9	[7]
003	-39.6	37.4	32.7	147.8	[7]
004	-52.5	77.5	23.7	171.4	[7]
005	-27.4	37.9	27.5	135	[7]
007	-29.7	19.3	74.3	175.2	[7]
008#	-60.3	30.1	45.2	168.1	[8]
009	-35.6	53.1	27.5	179	[7]
010	-34	49	39	65.9	[8]
013	-28	31	47	135	[8]
014*	-38	44	26	135	
015#	-53.2	48.5	60.4	150.8	[8]
016	-3	6	63	135	[8]
017#	-44.9	32.3	72.6	168.1	[8]

***eTable 2**. Target x, y, and z coordinates and coil orientation used for treatment, are listed along with references for e-field method used for that participant. *Note that participant 014 did not have an MRI and therefore targeting was based on MNI coordinates and a default 135-degree orientation. # indicates the coil coordinates at the scalp location.*

<u>1.8. Resting Motor Threshold and Additional Neurophysiology</u>:

Following their neuroimaging visit and before treatment initiation, participants underwent a neurophysiology session to determine their resting motor threshold (RMT) and to collect additional data for comparative analyses. RMT was measured from the abductor pollicis brevis (APB) muscle of the right hand, contralateral to the left hemisphere where TMS stimulation was applied. Participants were provided with earplugs and rested their right arm on a pillow, with instructions to keep their hand relaxed throughout the procedure. Using a MagVenture R30 stimulator with a CB60 coil (MagVenture A/S, Farum, Denmark), researchers administered single pulses to the motor cortex, ensuring the coil was flush against the scalp. The motor hotspot was located by identifying the site producing the largest visually detected motor-evoked potentials (MEPs) in the APB muscle on the thumb with minimum stimulation intensity. Once the hotspot was identified, two researchers independently determined the threshold intensity level using the Adaptive Parameter Estimation by Sequential Testing (PEST) algorithm [11] through the TMS Motor Threshold Assessment Tool, MTAT 2.0 software (http://www. clinicalresearcher.org/software.htm). The accepted RMT value was the lower of the two measurements. If there was a discrepancy of more than four percent between the two values, a third researcher conducted an additional RMT assessment to verify the selected lower value. Additional resting-state

© 2025 Appelbaum LG et al. *JAMA Network Open*.

electroencephalography (EEG) and concurrent TMS and EEG measures were collected for future retrospective analyses.

1.9. Accelerated Intermittent Theta Burst Treatment:

The accelerated TMS schedule used in the trial was designed according to the procedures reported by Cole and colleagues [12]. Patients were randomized to receive ten sessions per day of imaging-guided active or sham aiTBS at 90% resting motor threshold. Fifty intermittent theta-burst stimulation (iTBS) sessions (1,800 pulses per session, 50-min interval) were delivered in ten daily sessions over five consecutive days, for a total of 90,000 pulses throughout the treatment course. Stimulation was delivered to left DLPFC using coordinates calculated from fMRI connectivity analysis [7] with the coil orientation optimized using electric-field modeling [9]. All aiTBS was delivered at the UCSD Interventional Psychiatry Clinic with a physician on site.

All participants underwent a standardized setup procedure using the BrainSight neuronavigation system (Rogue Research, Montreal, Quebec). This optical tracking system, comprising a light-emitting camera and light-reflecting optical markers attached to the participant's head and the TMS coil, enabled precise localization of the coil in relation to the participant's head. Individualized target coordinates and orientation vectors for L-DLPFC treatment targets were loaded into the system along with the participant's reconstructed scalp and cortical surfaces from the T1 image.

Landmarks such as the nasion, the tip of the nose, and the left and right pre-auricular points were configured, labeled, and selected on the skin reconstruction. The registration involved aligning these landmarks between the BrainSight digital reconstruction and the participant's head while they wore a head strap mounted tracker. Validation of this alignment was conducted to confirm that the distance from the pointer crosshair to the scalp was within 3 mm. Calibration of the A/P coil in BrainSight was performed every morning prior to treatment using the calibration block. Throughout the treatment day, calibration was reperformed if the coil tracker moved more than 3 mm.

When calculated over all treatment sessions for 12 of the 13 participants (data did not save for one participant), the average targeting error was 1.23 mm, and the average coil orientation error was 15.86 degrees. For four participants, treatment was delivered at a fixed 135-degree orientation because they either did not have an MRI (n=1) or the orientation information was not available at the outset of treatment (n=3).

For all treatments, a Magventure Magpro R30 or X100 stimulator and Magventure Cool-B65 Active/Placebo Coil was used. A randomization treatment assignment number, provided by unblinded research personnel, was assigned to each participant and entered in the stimulator prior to treatment. Through a position sensor in the coil, the research software directed the operator as to which side of the coil (active or sham) should be angled towards the participant. Earplugs were provided to all participants for hearing protection. iTBS treatment consisted of 60 cycles of 10 biphasic bursts with three pulses each for a total of 1800 pulses per session at an inter-pulse interval of 20 ms. Each train of bursts was delivered at 5 Hz in a 2-second train with an 8-second intertrain interval. Treatment was delivered at 90% of the

© 2025 Appelbaum LG et al. *JAMA Network Open*.

participant's resting motor threshold. The treater performed a gradual intensity ramp-up, adjusting to patient discomfort levels as needed. All participants reached treatment intensity in at least 45 out of 50 sessions except for two patients who experienced tolerability issues throughout the treatment week (reaching their stimulation intensity in 68% and 38% of their total sessions, respectively). The sham treatment delivered an identical stimulation protocol but with the shielded side of the coil towards the participant. Somatosensory matched sham stimulation was achieved by placing two electrodes on the left side of the forehead under the coil for all patients. Electrical stimulation, scaled to the magnetic stimulator output intensity, was delivered synchronously with the sham magnetic pulses to mimic the sensation of magnetic stimulation. To assess blinding, patients were asked to guess the treatment allocation following the first and last treatment session.

1.10. Statistical Analysis:

The primary outcomes were MADRS scores at the pre-treatment screening and 1-week follow up, with additional consideration of the 4-week follow up scores. The MADRS was also administered at all five aiTBS sessions using a 24-hour retrospective timeframe. Treatment response status was defined on percent change between pre-treatment baseline and at the post-aiTBS follow-ups, with responders exhibiting a >50% reduction in MADRS scores at the 1-week follow-up timepoint.

Robust linear mixed effects modeling (rLME) was implemented using *robustlmm*, which applies robust estimating equations (i.e., Huber loss functions) using an iterative reweighting algorithm, to test the effects of time, treatment group (sham/active), and their interaction, with random intercept for participants, on MADRS scores, and posthoc comparisons performed using *emmeans*. Within-group analyses compared baseline and post-aiTBS follow-up MADRS scores separately within sham and active treatment groups (i.e., two "families" of hypothesis tests), with *p*-value correction using the Dunnett adjustment ("dunnettx"). Between-group analyses compared scores between treatment groups at baseline screening, 1- and 4-week follow-up visits (i.e., one "family" of hypothesis tests; no *p*-value adjustment applied). Effect sizes (Cohen's *d*) were computed for all contrasts, as well as 95% confidence intervals (CI). Hypothesis tests were two-sided, with alpha=0.05. One patient was lost to follow-up at the 4-week post-aiTBS visit.

1.11. Blinding

Both active and sham stimulation conditions used the Magventure Cool B65 A/P coil. In the sham configuration, the TMS coil was oriented with the shielded side towards the patient, projecting the active field away from the patient's head. Using an internal gyroscope, the device automatically detected the orientation of the coil and restricted operation until the coil was correctly positioned. In bother configurations, the electric sham stimulator was activated to induce a somatosensory-matched sham stimulation that was delivered through electrodes placed on the scalp around the target area. This placebo therefore delivered brief electric pulses to the scalp simultaneously with the TMS pulses. The intensity of these electric pulses was set to match the sensation of active treatment. This calibration was

© 2025 Appelbaum LG et al. *JAMA Network Open*.

especially important for participants who had previously received TMS, enabling the blinding to remain effective for all participants.

To test the quality of this blind, participants were surveyed to assess whether they believed they were receiving active or sham stimulation at two time points during their treatment week, after the very first stimulation session (i.e., Day 1, Session 1) and the very last stimulation session (Day 5, Session 50). At these times, they were asked “Do you think you are receiving active or sham stimulation?” and “on a scale from 1 (low confidence) to 5 (high confidence), how confident are you in this answer?” Data for these questions is available for 9 of the 13 participants.

While this sample is not sufficient to perform a statistical test of the blind, the data from the post-treatment (session 50) are presented in **eTable 3**. As depicted, five of the nine participants with data correctly identified the type of stimulation they received, while four participants guessed incorrectly. Though not definitive, these data support the conclusion that the blinding was effective; participants were not able to reliably distinguish between the active and sham treatments.

	Do you think you are receiving active or sham?	How confident are you in your rating?	Treatment arm	Match
BIP002	Active	3	Sham	
BIP003	Sham	4	Sham	1
BIP004	Sham	2	Sham	1
BIP005	Active	3	Sham	
BIP013	Sham	3	Sham	1
BIP014	Active	4	Active	1
BIP015	Sham	3	Active	
BIP016	Active	3	Sham	
BIP017	Sham	4	Sham	1

***eTable 3**. Guesses, confidence, and actual treatment assignments for nine participants with whom blinding data were available.*

eReferences

1. Montgomery SA, Asberg M. Anewdepression scale designed to be sensitive to change. *Br J Psychiatry.* 1979(134):382-389.

2. Sheline YI, Makhoul W, Batzdorf AS, et al. Accelerated Intermittent Theta-Burst Stimulation and Treatment-Refractory Bipolar Depression: A Randomized Clinical Trial. *JAMA Psychiatry.* 2024;81(9):936-941.

© 2025 Appelbaum LG et al. *JAMA Network Open*.

3. Sackeim HA, Aaronson ST, Bunker MT, et al. The assessment of resistance to antidepressant treatment: Rationale for the Antidepressant Treatment History Form: Short Form (ATHF-SF). *J Psychiatr Res.* 2019;113:125-136.

4. Tavares DF, Suen P, Rodrigues Dos Santos CG, et al. Treatment of mixed depression with theta-burst stimulation (TBS): results from a double-blind, randomized, sham-controlled clinical trial. *Neuropsychopharmacology.* 2021;46(13):2257-2265.

5. Tondo L, Vazquez G, Baldessarini RJ. Mania associated with antidepressant treatment: comprehensive meta-analytic review. *Acta Psychiatr Scand.* 2010;121(6):404-414.

6. Hedeker D, Gibbons, R.D., Waternaux, C.,. Sample Size Estimation for Longitudinal Designs with Attrition: Comparing Time-Related Contrasts Between Two Groups. *Journal of Educational and Behavioral Statistics.* 1999;24(1):70-93.

7. Cash RFH, Cocchi L, Lv J, Wu Y, Fitzgerald PB, Zalesky A. Personalized connectivity-guided DLPFC-TMS for depression: Advancing computational feasibility, precision and reproducibility. *Hum Brain Mapp.* 2021;42(13):4155-4172.

8. Balderston NL, Beer JC, Seok D, et al. Proof of concept study to develop a novel connectivity-based electric-field modelling approach for individualized targeting of transcranial magnetic stimulation treatment. *Neuropsychopharmacology.* 2022;47(2):588-598.

9. Dannhauer M, Huang Z, Beynel L, Wood E, Bukhari-Parlakturk N, Peterchev AV. TAP: targeting and analysis pipeline for optimization and verification of coil placement in transcranial magnetic stimulation. *J Neural Eng.* 2022;19(2).

10. Blumberger DM, Vila-Rodriguez F, Thorpe KE, et al. Effectiveness of theta burst versus high-frequency repetitive transcranial magnetic stimulation in patients with depression (THREE-D): a randomised non-inferiority trial. *Lancet.* 2018;391(10131):1683-1692.

11. Awiszus F. TMS and threshold hunting. *Suppl Clin Neurophysiol.* 2003;56:13-23.

12. Cole EJ, Phillips AL, Bentzley BS, et al. Stanford Neuromodulation Therapy (SNT): A Double-Blind Randomized Controlled Trial. *Am J Psychiatry.* 2022;179(2):132-141.

© 2025 Appelbaum LG et al. *JAMA Network Open*.
