# Supplementary material for: Accelerated Intermittent Theta-Burst Stimulation for Treatment-Resistant Bipolar Depression: A Randomized Clinical Trial
Source: JAMA Netw Open. 2025 Feb 11;8(2):e2459361. doi: 10.1001/jamanetworkopen.2024.59361 (PMC11815521; doi:10.1001/jamanetworkopen.2024.59361)
Supplement: Supplement 3. — Data Sharing Statement [file jamanetwopen-e2459361-s003.pdf]

Data Sharing Statement

Appelbaum. Accelerated Intermittent Theta-Burst Stimulation for Treatment-Resistant Bipolar Depression. *JAMA Netw Open*. Published February 11, 2025.
doi:10.1001/jamanetworkopen.2024.59361

Data
Additional Information: Bipolar Efficacy Biomarkers for rTMS, NCT05393648, https://clinicaltrials.gov/study/NCT05393648
Data available: Yes
Data types: Deidentified participant data
How to access data: Data requests can be made to the corresponding author, Dr. Greg Appelbaum at greg@health.ucsd.edu
When available: With publication

Supporting Documents
Document types: None

Additional Information
Who can access the data: anyone requesting the data
Types of analyses: for any purpose
Mechanisms of data availability: with a signed access agreement
